# Direct Sequencing of 5‐Methylcytosine and 5‐Hydroxymethylcytosine at Single‐Base Resolution Unravels Their Distinct Roles in Alzheimer's Disease

**DOI:** 10.1002/advs.202507843

**Published:** 2025-07-16

**Authors:** Zi‐Xin Wang, Faying Chen, Bao‐Dan He, Fan‐Chen Wang, Jiaxue Cha, Yu Song, Wei‐Ying Meng, Wan‐Yue Zou, Yu‐Tao Fu, Shu‐Xia Sun, Zhi‐Yan Sun, Hao‐Ming Jiang, Ke‐Yao Zhao, Yujun Hou, Jiejun Shi, Jian‐Huang Xue

**Affiliations:** ^1^ Key Laboratory of Spine and Spinal Cord Injury Repair and Regeneration of Ministry of Education Tongji Hospital affiliated to Tongji University Frontier Science Center for Stem Cell Research School of Life Sciences and Technology Tongji University Shanghai 200092 China; ^2^ Institute for Regenerative Medicine State Key Laboratory of Cardiology and Medical Innovation Center Shanghai East Hospital Shanghai Key Laboratory of Signaling and Disease Research Frontier Science Center for Stem Cell Research School of Life Sciences and Technology Tongji University Shanghai 200092 China

**Keywords:** 5‐hydroxymethylcytosine (5hmC), 5‐methylcytosine (5mC), Alzheimer's disease (AD), CMD1‐Deaminase sequencing (CD‐seq), CMD1‐TET bisulfite sequencing (CT‐seq)

## Abstract

Alzheimer's disease (AD) is associated with genetic risk factors and widespread epigenetic alterations. 5‐Hydroxymethylcytosine (5hmC), an oxidized derivative of 5‐methylcytosine (5mC), constitutes up to 20% of 5mC in neuronal DNA and is implicated in aging and neurodegeneration. However, the precise roles of DNA modifications in AD remain unclear, partly due to the lack of accurate detection methods. Here, two orthogonal sequencing methods are introduced: CMD1‐Deaminase sequencing (CD‐seq) and CMD1‐TET bisulfite sequencing (CT‐seq), which enable direct, independent detection of 5mC. When combined with APOBEC‐coupled epigenetic sequencing (ACE‐seq) or TET‐assisted bisulfite sequencing (TAB‐seq) for 5hmC mapping, these techniques provide base‐resolution, subtraction‐free profiling of DNA modifications. Applying them to hippocampal tissue from AD model mice, a significant reduction in 5hmC levels is identified without corresponding changes in 5mC, suggesting that 5hmC functions as an independent epigenetic mark in AD pathogenesis. These findings underscore the importance of precise 5mC/5hmC discrimination and suggest that 5hmC and its regulatory pathways may serve as potential therapeutic targets for AD.

## Graphical abstract

This study introduces two orthogonal sequencing methods, CMD1‐Deaminase sequencing (CD‐seq) and CMD1‐TET bisulfite sequencing (CT‐seq), that enable direct, independent detection of 5mC. Using these approaches together with ACE‐seq or TAB‐seq for 5hmC identification, the study reveals a significant reduction in 5hmC, but not 5mC, in the hippocampus of Alzheimer's disease (AD) model mice, supporting a role for 5hmC as an independent epigenetic mark in AD pathogenesis.

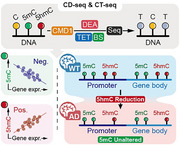

## 1 Introduction

Alzheimer's disease (AD) is a progressive neurodegenerative disorder characterized by amyloid‐beta (Aβ) plaque deposition and tau neurofibrillary tangles in the brain, leading to cognitive decline, memory loss, and impaired daily functioning.^[^ ^1^, ^2^ ^]^ Risk factors for AD include aging, genetic variants, and lifestyle.^[^ ^3^, ^4^ ^]^ Additionally, epigenetic modifications, particularly DNA modifications, have been strongly implicated in AD pathogenesis.^[^ ^5^ ^]^ Among these, 5‐methylcytosine (5mC) and its oxidized derivative 5‐hydroxymethylcytosine (5hmC) are exceptionally abundant in the brain, where they dynamically regulate neurodevelopment and synaptic plasticity.^[^ ^6^, ^7^ ^]^ However, the precise distribution and functional roles of 5mC and 5hmC in AD progression remain poorly understood.

As the best‐studied DNA modification, 5mC is closely associated with transcriptional regulation and plays critical roles in various biological processes in mammals, including X chromosome inactivation, genomic imprinting, and transposon suppression.^[^ ^8^, ^9^ ^]^ In mammalian cells, 5mC is generated by DNA methyltransferase (DNMT) family proteins, while the demethylation process involves ten‐eleven translocation (TET) dioxygenases.^[^ ^10^, ^11^ ^]^ TET proteins belong to the α‐ketoglutarate‐ and Fe(II)‐dependent dioxygenase superfamily and are capable of converting 5mC into 5hmC, 5‐formylcytosine (5fC), and 5‐carboxylcytosine (5caC). The latter two can be excised by thymine DNA glycosylase (TDG) to initiate the base excision repair (BER) pathway, restoring the unmodified state and resulting in active DNA demethylation.^[^ ^12^, ^13^, ^14^ ^]^ Moreover, oxidation of 5mC disrupts its interaction with ubiquitin like with PHD and ring finger domains 1 (UHRF1), a core factor in the maintenance of DNA methylation, leading to passive DNA demethylation.^[^ ^15^, ^16^ ^]^

The abundance of 5hmC, 5fC, and 5caC varies significantly across different cell types. While 5fC and 5caC are typically present at low or undetectable levels in most cells, 5hmC can constitute up to 20% of 5mC in neurons.^[^ ^13^, ^17^ ^]^ This suggests that 5hmC, rather than 5fC or 5caC, may play a more prominent role in gene expression regulation, in addition to serving as an intermediate in DNA demethylation.^[^ ^18^, ^19^, ^20^, ^21^ ^]^ Indeed, 5hmC has been shown to be enriched in gene bodies and associated with actively transcribed genes.^[^ ^22^, ^23^, ^24^, ^25^, ^26^ ^]^ Another study indicated that 5hmC limits the magnitude of gene expression changes during aging,^[^ ^17^ ^]^ further supporting its role as an epigenetic mark independent of 5mC. However, the biological functions and underlying mechanisms of 5hmC in brain development and neurodegenerative disorders, such as AD, remain largely unexplored.^[^ ^27^ ^]^ Previous studies have yielded inconsistent findings regarding the dynamics of 5mC and 5hmC in AD,^[^ ^28^, ^29^, ^30^, ^31^, ^32^, ^33^, ^34^ ^]^ in part due to the lack of accurate, single‐base resolution analysis of these modifications in AD contexts.

Accurately mapping 5hmC is crucial for studying its biological functions. Since bisulfite sequencing (BS‐seq), the gold standard for detecting 5mC, fails to distinguish 5hmC from 5mC, several methods have been developed to map 5hmC or 5mC, either dependent on or independent of BS‐seq (Figure S1A, Supporting Information).^[^ ^35^, ^36^ ^]^ The first strategy to map 5hmC at single‐base resolution is TET‐assisted bisulfite sequencing (TAB‐seq).^[^ ^37^ ^]^ In this method, TET converts 5mC into 5caC, which is read as thymine (T) after BS‐seq, while 5hmC resists this conversion and remains as C after sequencing due to its glucosylation by β‐glucosyltransferase (βGT) (Figure S1B, Supporting Information). Due to the DNA damage caused by bisulfite treatment, the APOBEC‐coupled epigenetic sequencing (ACE‐seq) was developed as a bisulfite‐free alternative for detecting 5hmC (Figure S1C, Supporting Information).^[^ ^38^ ^]^ After identifying 5hmC, confident 5mC sites are typically inferred by subtracting 5hmC levels from whole‐genome bisulfite sequencing (WGBS) data.^[^ ^37^, ^39^ ^]^ However, due to insufficient sequencing depth, this subtraction approach can lead to an underestimation of 5mC, particularly at sites with high 5hmC abundance, potentially resulting in negative values for 5mC levels. Albeit several other methods have been proposed to detect either 5mC or 5hmC, parallel techniques for independently identifying both modifications are still lacking, especially those based on enzymes with high conversion rates and low DNA damage.

To address this challenge, we developed two orthogonal methods for the direct identification of 5mC by utilizing 5mC modification enzyme 1 (CMD1), which converts 5mC into 5‐glyceryl‐methylcytosine (5gmC) without affecting 5hmC.^[^ ^40^ ^]^ In CMD1‐Deaminase sequencing (CD‐seq), 5gmC is expected to resist deaminase activity, while 5hmC is fully converted, enabling the direct detection of 5mC. When combined with ACE‐seq, these techniques eliminate the need for subtraction, allowing for the independent detection of 5mC or 5hmC. Additionally, CMD1‐TET bisulfite sequencing (CT‐seq) can also convert 5hmC into T, leaving 5mC unaffected. CT‐seq can then be used alongside TAB‐seq to independently identify 5mC and 5hmC.

Using these methods, we generated comprehensive 5mC and 5hmC profiles in the hippocampus of wild‐type (WT) and AD model mice. Our analysis revealed a significant reduction in 5hmC levels in AD mice, while 5mC levels remained largely unchanged. Notably, 5mC was negatively correlated with gene expression, whereas 5hmC showed a positive correlation, regardless of its location in promoters or gene bodies. However, despite reduced 5hmC in AD, its impact on gene expression was limited, suggesting complex regulatory mechanisms beyond direct transcriptional control. These findings indicate that 5hmC functions as a critical, independent epigenetic mark in the brain and may play a key role in AD pathogenesis.

## 2 Results

### 2.1 Establishment of CD‐Seq

**Figure 1:**
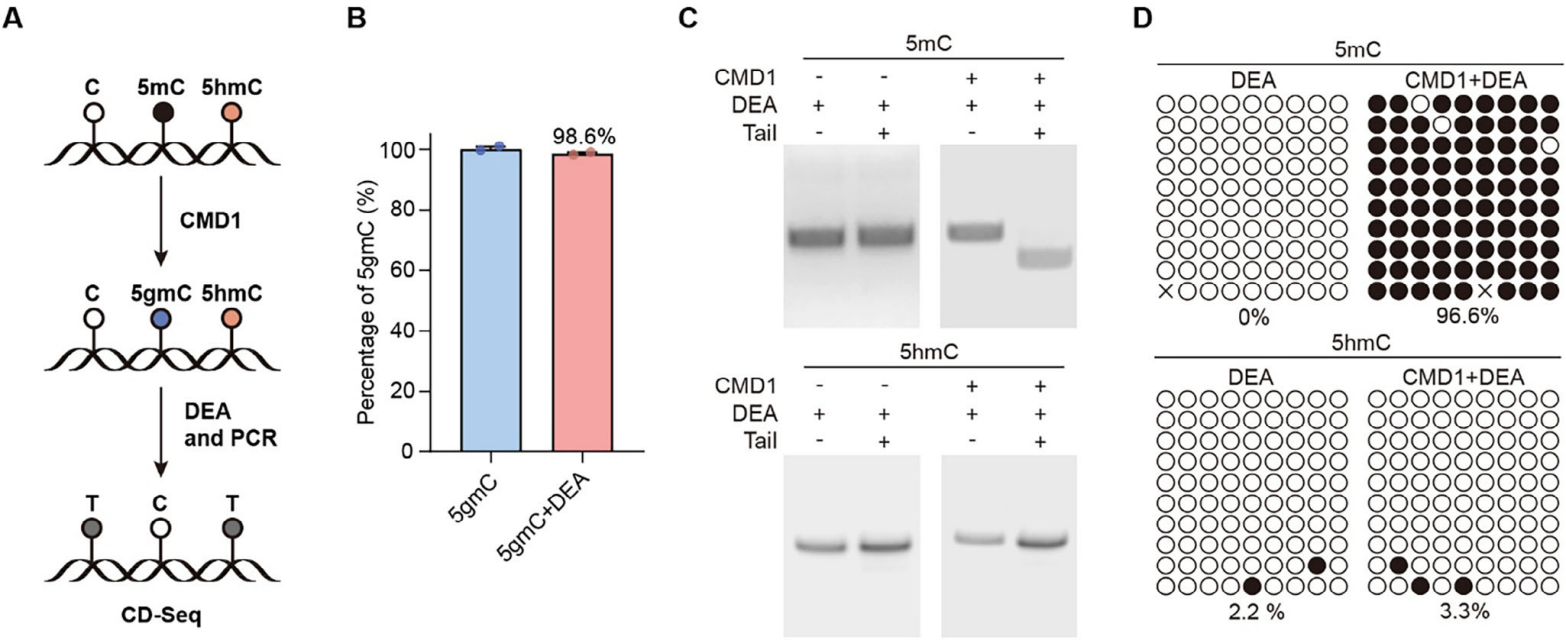
Establishment of CD‐seq for detecting 5mC in DNA. A) Schematic diagram illustrating the CD‐seq workflow for the detection of 5mC. B) Mass spectrometry analysis of 5gmC before and after DEA treatment. Data are presented as mean ± SD from two independent biological replicates. C) Restriction endonuclease digestion assay to detect cytosine modification variants in ACGT contexts after sequential CMD1 reaction, DEA treatment, and PCR amplification. Representative images from two independent biological replicates are shown. D) CD‐seq analysis of 5mC‐ or 5hmC‐DNA. Each circle represents a CpG site, with unfilled circles indicating converted cytosines (5mC without CMD1 treatment or 5hmC) and filled black circles representing unaltered ones (5mC with CMD1 treatment). The data shown are representative of two independent biological replicates. The 5mC‐ and 5hmC‐DNA used in these assays were prepared by PCR amplification, and the sequence is provided in the methods section.

Inspired by TAB‐seq and ACE‐seq (Figure S1B,C, Supporting Information), which specifically convert 5mC into T while protecting 5hmC through glucosylation,^[^ ^37^, ^38^ ^]^ we sought to identify a unique enzyme capable of protecting 5mC from deamination. Previously, we discovered a novel DNA oxygenase, CMD1, a TET homologue found in green algae.^[^ ^40^ ^]^ This enzyme transfers the glyceryl moiety of vitamin C (VC) to the methyl carbon of 5mC, resulting in the formation of 5gmC. The bulky glyceryl group of 5gmC endows it with the potential to resist deamination, similar to the protection afforded to 5hmC in TAB‐seq and ACE‐seq through β‐glucosylation. This property enables the direct detection of 5mC while 5hmC is fully deaminated. Based on this, we proposed CD‐seq, where 5mC is converted into 5gmC by CMD1, and the deamination of 5hmC by a specific deaminase allows for the differentiation between 5mC and 5hmC (**Figure** **1**A).

To test our hypothesis, we purified the CMD1 protein from *E. coli* (Figure S2A, Supporting Information) and confirmed its specific activity in generating 5gmC from 5mC, but not 5hmC, via mass spectrometry (MS) analysis (Figure S2B,C, Supporting Information). Simultaneously, we evaluated the catalytic activity of enzymes used in the TAB‐seq and ACE‐seq for detecting 5hmC. MS analysis showed that TET proteins efficiently converted both 5mC and 5hmC into 5fC or 5caC (Figure S3A, Supporting Information). Additionally, 5hmC was modified by T4 Phage βGT, whereas 5mC was minimally affected (Figure S3B, Supporting Information). For cytosine deamination, we tested a commercial DNA deaminase mix (DEA) instead of APOBEC3A.^[^ ^41^ ^]^ Remarkably, almost all C, 5mC, and 5hmC could be efficiently converted, confirming the potential of DEA in the direct identification of DNA modifications (Figure S3C, Supporting Information). Compared to BS‐seq, which cannot distinguish between 5mC and 5hmC (Figure S3D, Supporting Information), both TAB‐seq and ACE‐seq can directly identify 5hmC (Figure S3E,F, Supporting Information). However, based on our results, TAB‐seq misidentified ≈2% of 5mC as potential false positives for 5hmC (Figure S3A,E, Supporting Information). In contrast, ACE‐seq exhibited a higher 5mC conversion rate and was thus used as the primary method for 5hmC identification in this study (Figure S3C,F, Supporting Information).

In contrast to the deamination of 5hmC, 5gmC completely inhibited the activity of DEA (Figure 1B). This inhibition allowed CD‐seq to directly identify 5mC, as corroborated by restriction enzyme analysis and Sanger sequencing (Figure 1C,D). Therefore, CD‐seq is a reliable approach for genome‐wide 5mC profiling. When combined with ACE‐seq, 5mC and 5hmC can be independently and directly determined, obviating the need for a subtraction process.

### 2.2 Application of CD‐Seq in Genome‐Wide 5mC Profiling

To further validate the reliability of CD‐seq for profiling 5mC, we examined its distribution in mouse embryonic stem cells (ES) and compared the sequencing results with previously reported 5mC landscapes. TAPSβ (TET‐assisted pyridine borane sequencing β) has been shown to specifically convert 5mC into 5caC, which is subsequently reduced by pyridine borane to dihydrouracil (DHU). After PCR and sequencing, DHU is read as T, while 5hmC remains as C due to the protective effect of glucosylation, enabling specific readout of 5mC.^[^ ^42^ ^]^ The published TAPSβ data were downloaded for comparison with the CD‐seq dataset (Figure S4, Supporting Information).

The CD‐seq metrics confirmed the high quality of the data. Spike‐in results showed that ≈97% of 5hmC was read as T after CD‐seq, while over 92% of 5mC remained unaltered and were directly identified (Figure S4A, Supporting Information). This demonstrated CD‐seq's ability to efficiently distinguish between 5mC and 5hmC. Comparison with TAPSβ revealed a similar DNA methylation pattern across all genomic contexts (Figure S4B, Supporting Information). A slightly higher methylation level was detected by CD‐seq, which could be attributed to incomplete conversion of 5hmC in CD‐seq or limited identification of 5mC in TAPSβ. Nevertheless, CD‐seq showed a high degree of consistency with TAPSβ across various genomic contexts, and a substantial overlap was observed between the 5mC sites identified by CD‐seq and TAPSβ (Figure S4C–G, Supporting Information). Representative genomic loci also exhibited comparable 5mC levels between these different methods (Figure S4H–K, Supporting Information), validating its use for genome‐wide 5mC profiling at single‐base resolution.

### 2.3 Genome‐wide Profiling of 5mC and 5hmC in the Hippocampus of AD Model Mice

**Figure 2:**
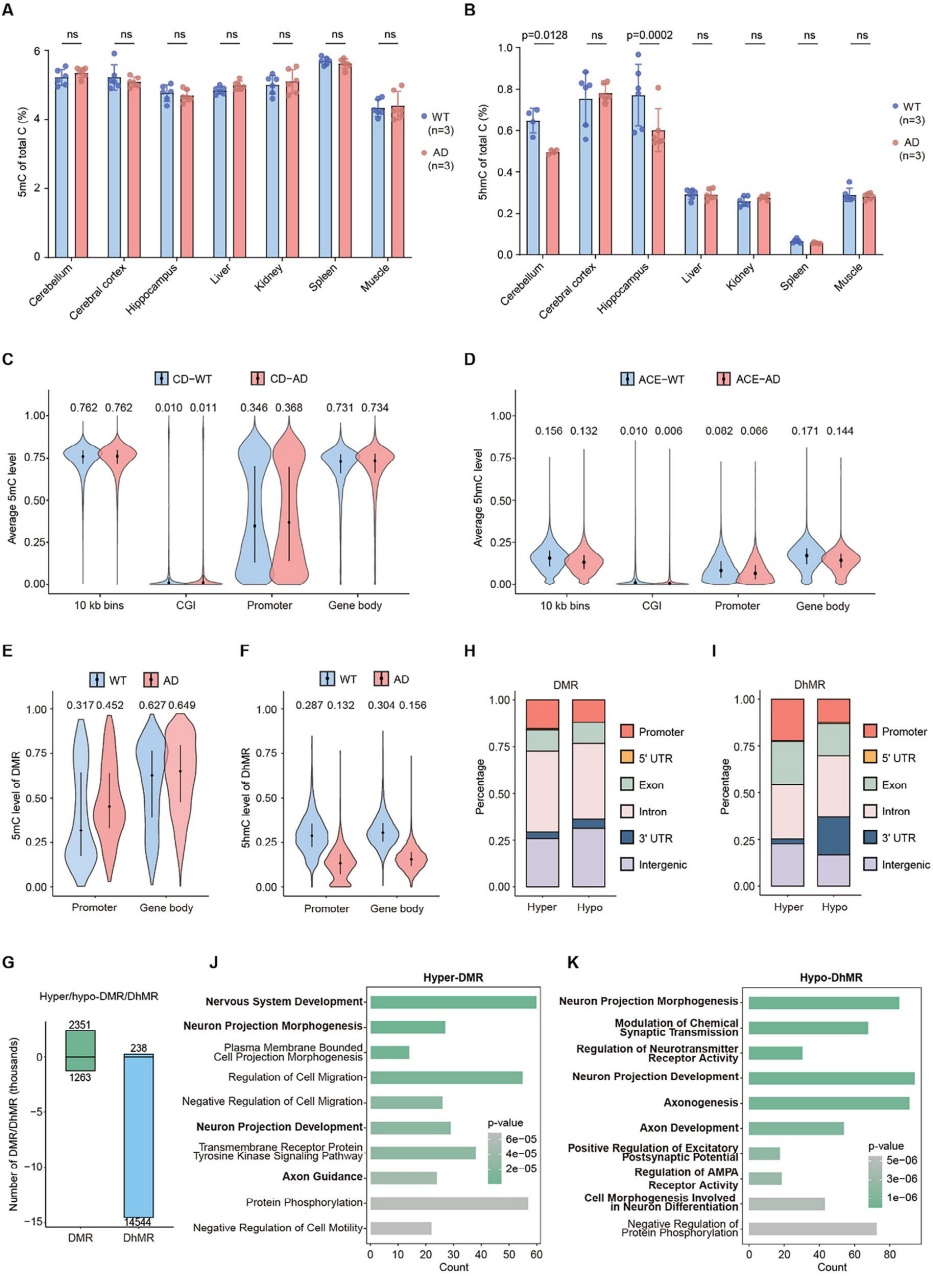
Independent analysis of 5mC and 5hmC by CD‐seq and ACE‐seq reveals global changes of 5hmC in AD. A,B) Mass spectrometry analysis of 5mC (A) and 5hmC (B) levels in various mouse tissues. Blue bars represent WT samples, and red bars represent AD samples (*n* = 3). Data are presented as mean ± SD from three independent biological replicates. *P‐*values were determined by two‐way ANOVA. Statistical significance was defined as *p* < 0.05; ns indicates not significant. Both WT and AD mice used were 15 months old, and the entire hippocampus was collected for genomic DNA extraction and downstream analyses. C,D) Average modification levels of 5mC detected by CD‐seq (C), and 5hmC detected by ACE‐seq (D) across different genomic contexts for WT (blue) and AD (red) samples, based on all detected CpG sites. The region ±1 kb from the transcription start site is defined as the promoter. The width of the violin plot represents the frequency of data at each value. The short horizontal line denotes the interquartile range of the data, while the black dot indicates the median. Specific numerical values are annotated for clarity. All the sequencing data were analyzed based on two independent biological replicates, which are summarized in Figure S6A. E,F) Average 5mC levels of DMR (E) detected by CD‐seq, and 5hmC level of DhMR (F) detected by ACE‐seq in WT (blue) and AD (red) samples. DMR/DhMR are defined as regions spanning ≥ 10 CpGs, with a mean modification difference ≥ 0.1 and a MWU‐test *p* value < 0.001. The width of the violin plot represents the frequency of data at each value. The short horizontal line denotes the interquartile range of the data, while the black dot indicates the median. Specific numerical values are annotated for clarity. G) Number of hyper‐DMR, hypo‐DMR, hyper‐DhMR, and hypo‐DhMR. Regions with increased modification levels in AD are classified as hyper‐DMR/DhMR, and those with decreased modification levels are classified as hypo‐DMR/DhMR. H,I) Distribution of hyper/hypo‐DMR (H) and hyper/hypo‐DhMR (I) across different genomic contexts. J,K) Gene Ontology analysis of genes with hyper‐DMR detected by CD‐seq (J) and hypo‐DhMR detected by ACE‐seq (K). The color of each bar indicates the *p*‐value, with darker shades representing lower *p* values.

We then utilized the 5×FAD mouse model, a canonical model of AD, harboring five AD‐linked mutations associated with familial Alzheimer's disease,^[^ ^43^ ^]^ to investigate the dynamics of 5mC and 5hmC in different tissues of AD mice. Both WT and 5×FAD mice at 15 months of age were analyzed, and amyloid pathology was confirmed in the AD group (Figure S5A, Supporting Information). Genomic DNA was subsequently extracted from multiple tissues of WT and AD mice for epigenetic profiling. Our results showed that 5mC levels were comparable across tissues in both WT and AD mice (**Figure** **2**A). However, MS analysis revealed a significant reduction in 5hmC levels in the brains of AD mice, particularly in the hippocampus and cerebellum (Figure 2B). Interestingly, hippocampal 5hmC levels are initially similar between WT and AD mice at 5 months of age. However, the degree of differential 5hmC increases progressively with age and disease progression in AD model mice compared to WT controls. (Figure S5B,C, Supporting Information). Since hippocampal neurons play a crucial role in encoding and consolidating new memories and are severely affected in AD,^[^ ^44^ ^]^ we subsequently employed CD‐seq and ACE‐seq to map the distribution of 5mC and 5hmC across the hippocampal genome of 15‐month‐old mice (Figure S6A, Supporting Information), aiming to investigate the relationship between these DNA modifications and the progression of AD.

Consistent with previous studies, most 5mC sites were located in CpG regions;^[^ ^45^ ^]^ however, we also detected 5mC at CHG and CHH sites (Figure S6B, Supporting Information). The distribution of 5hmC mirrored that of 5mC in both WT and AD samples (Figure S6C, Supporting Information). Biological replicates of two WT and AD mice yielded consistent results for CD‐seq, although the overlap of 5hmC sites between replicated samples in ACE‐seq was lower, likely due to the lower abundance of 5hmC (Figure S6D–I, Supporting Information). The average modification levels of 5mC and 5hmC across various genomic contexts were calculated (Figure S7A,B, Supporting Information). In line with MS data, 5mC levels in the hippocampus of AD mice did not differ significantly from those in WT mice, with only a slight increase of 5mC in the promoters (Figure 2C). In contrast, 5hmC was substantially reduced in AD mice, particularly at genomic regions across gene bodies, and to a lesser content in promoters (Figure 2D; Figure S7A,B, Supporting Information), indicating distinct changes of 5mC and 5hmC across various genomic contexts. Interestingly, 5hmC levels showed a positive correlation with 5mC in promoters; however, this correlation was absent in gene body regions (Figure S7C,D, Supporting Information), suggesting a complex and context‐dependent relationship between 5mC and 5hmC across the genome.

We next characterized the differentially methylated regions (DMR) and differentially hydroxymethylated regions (DhMR) in the promoters and gene bodies of WT and AD mice and assessed the average modification ratios for each DNA mark. As expected, 5hmC levels were significantly decreased in DhMR, while 5mC showed an increase in DMR (Figure 2E,F). Specifically, only 1.6% of DhMR were hypermodified at DhMR, with the majority of these sites in AD mice exhibiting a loss of DNA hydroxymethylation. In contrast, more DMR were hypermethylated rather than hypomethylated (Figure 2G). Many DMR were located in intronic regions, while hypo‐DMR were also enriched in intergenic regions (Figure 2H). Notably, a larger proportion of gene bodies, including untranslated regions (UTR) lost 5hmC in AD mice compared to promoters, indicating a specific regulatory role of 5hmC in gene bodies during AD pathogenesis (Figure 2I).

Functionally, hyper‐DMR and hypo‐DhMR were enriched in pathways related to neurogenesis or associated regulatory functions, in contrast to hypo‐DMR and hyper‐DhMR, emphasizing the crucial roles of 5hmC loss and 5mC gain in AD pathogenesis (Figure 2J,K; Figure S7E,F, Supporting Information). In addition, we characterized differential methylated genes (DMG) and differential hydroxymethylated genes (DhMG) as genes containing DMR or DhMR, with the intersection group termed D(M/hM)G (Figure S7G, Supporting Information). Notably, only DhMG and D(M/hM)G were enriched in neuronal functions (Figure S7H–J and Table S1, Supporting Information), further supporting the critical role of 5hmC in AD, rather than 5mC.

### 2.4 5hmC Decreases Significantly in AD Without Altering 5mC Levels

**Figure 3:**
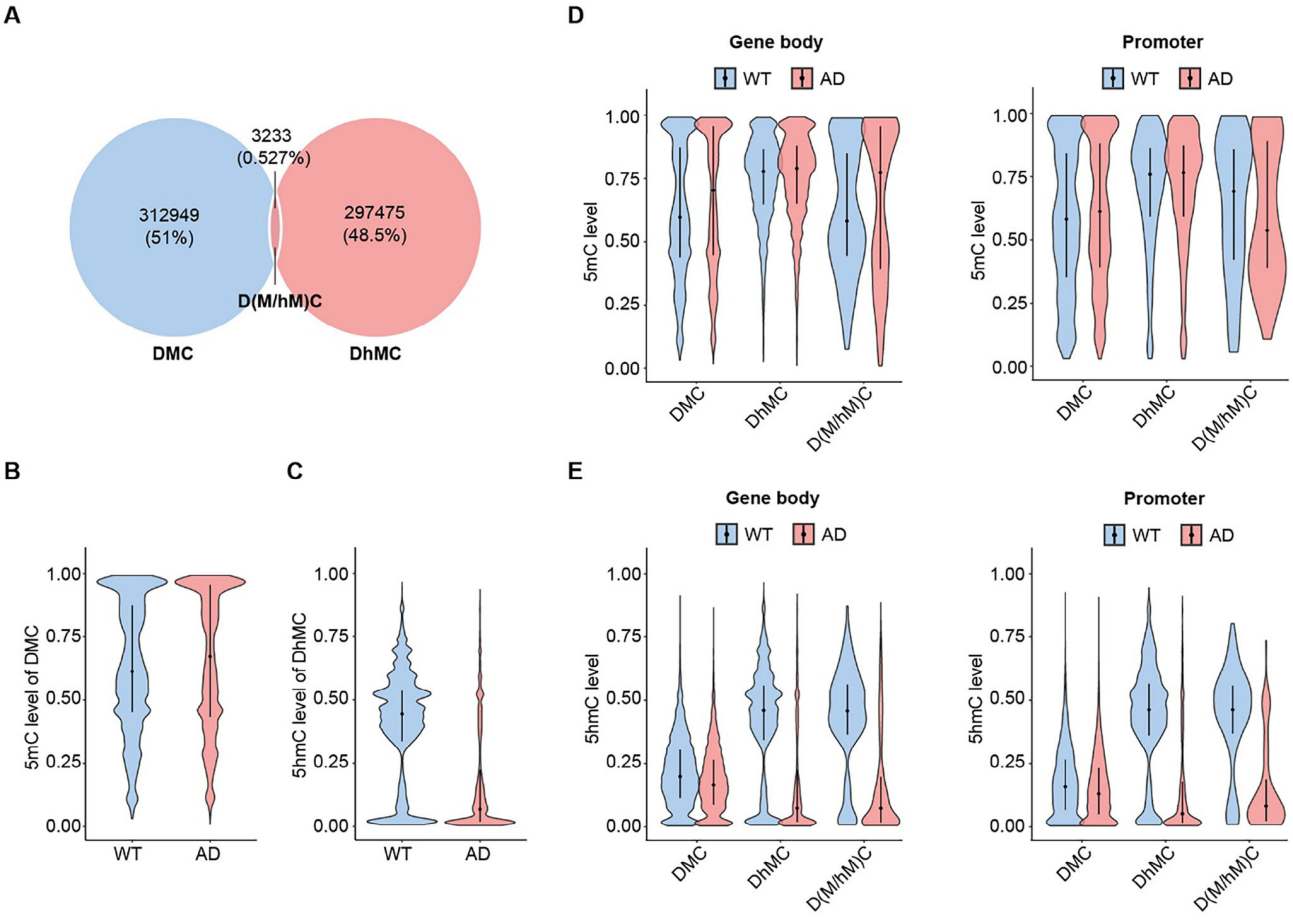
Independent analysis of 5mC and 5hmC reveals exclusive distribution of DMC and DhMC. A) Overlap analysis between DMC and DhMC. Cytosines in the overlapping region are classified as D(M/hM)C. DMC and DhMC with a *p* value < 0.05 and a mean modification difference ≥ 0.1 were considered significantly differentially methylated or hydroxymethylated. B,C) Average levels of 5mC in DMC (B) or 5hmC in DhMC (C) in WT and AD samples. The width of the violin plot represents the frequency of data at each value. The short horizontal line denotes the interquartile range of the data, while the black dot indicates the median. D,E) 5mC (D) or 5hmC (E) levels of all DMC, DhMC, and D(M/hM)C within gene bodies (left) and promoters (right) in WT (blue) and AD (red) mice. The width of the violin plot represents the frequency of data at each value. The short horizontal line denotes the interquartile range of the data, while the black dot indicates the median.

To further investigate the base‐resolution relationship between 5mC and 5hmC across different genomic contexts, we categorized the differentially modified cytosines into three groups: differentially methylated cytosines (DMC), differentially hydroxymethylated cytosines (DhMC), and the intersecting group (D(M/hM)C), which includes sites showing changes in both 5mC and 5hmC. Notably, the overlapping sites exhibiting simultaneous changes in both 5mC and 5hmC were relatively limited (**Figure** **3**A). Within the D(M/hM)C group, the loss of 5hmC was often associated with either an increase or decrease in 5mC levels at these loci. In contrast, the DhMC group, which exhibited stable 5mC levels, strongly suggested that the dynamics of 5hmC occur independently of 5mC changes.

Furthermore, we quantified the average levels of 5mC and 5hmC within each DMC and DhMC, revealing a significant reduction in 5hmC and an increase in 5mC in AD model mice (Figure 3B,C). We then investigated locus‐specific changes in 5hmC and 5mC across various genomic contexts. In DMC, 5mC levels showed an increase in both gene bodies and promoters, accompanied by a slight decrease in 5hmC. In contrast, in DhMC, 5hmC levels were notably reduced, while 5mC remained unchanged. Interestingly, in the D(M/hM)C group, 5hmC decreased significantly in both gene bodies and promoters, with 5mC increasing in gene bodies but decreasing in promoters (Figure 3D,E). These findings suggest that 5hmC may facilitate 5mC demethylation at specific loci in gene bodies, while acting as an independent epigenetic mark in most of other genomic regions.

### 2.5 Reduction of 5hmC is not Associated with Gene Expression Changes in AD

**Figure 4:**
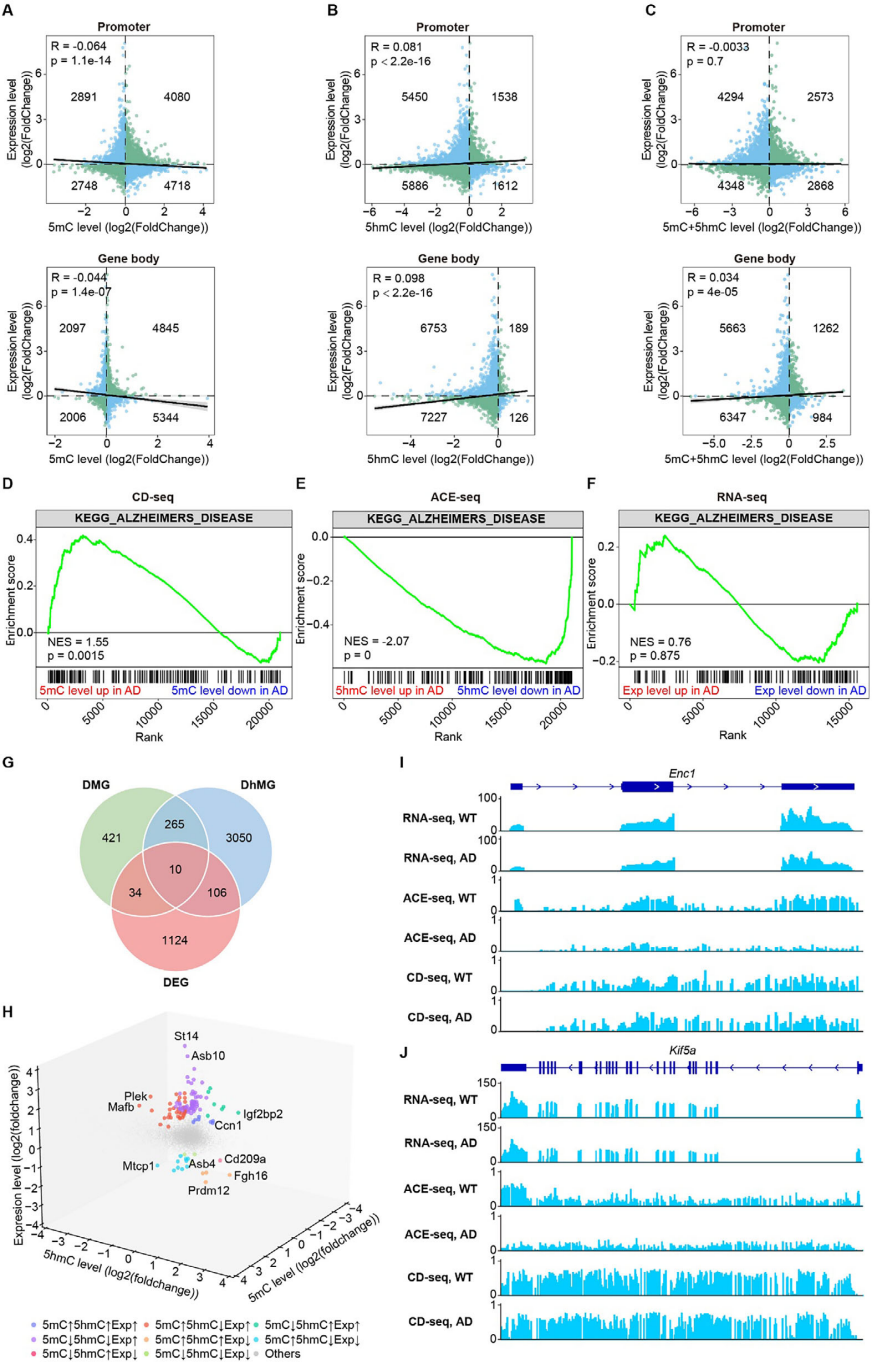
5hmC positively correlates with gene expression. A–C) Correlation between gene expression and the levels of 5mC (A), 5hmC (B), and 5mC+5hmC (C) in the promoter (top) and gene body (bottom) regions. Each point represents a gene; green points indicate a positive correlation, while blue points indicate a negative correlation. D–F) Gene set enrichment analysis (GSEA) of AD‐related genes in CD‐seq (D), ACE‐seq (E), and RNA‐seq (F). The green line indicates the normalized enrichment score (NES) across the gene ranks. The bar at the bottom shows the changes in 5mC levels, 5hmC levels, or gene expression levels, respectively, among the genes. G) Overlap analysis of DMG, DhMG, and DEG. DMG and DhMG are defined as genes with DMR or DhMR, while DEG refers to genes with a *p* value < 0.05 and fold change > 1.5 in AD samples compared with WT. H) 3D scatter plot showing the relationship between 5mC or 5hmC levels in promoters and gene expression. Each point represents a gene, with the color indicating the changes in 5mC, 5hmC, and gene expression levels. I,J) Genome browser views showing the expression, methylation, and hydroxymethylation levels of representative genes: *Enc1* (ectodermal‐neural cortex 1, chr13:97,369,361‐97,442,213) (I) and *Kif5a* (kinesin family member 5A, chr10:127,044,139‐127,201,474) (J). For CD‐seq and ACE‐seq, each vertical bar represents a CpG site.

DNA modifications are crucial regulators of gene expression. While 5mC in promoters is generally considered a repressive mark for transcription,^[^ ^46^ ^]^ 5hmC has been shown to correlate with active gene transcription.^[^ ^23^, ^24^, ^25^, ^26^ ^]^ Consistent with this, our results indicate that 5mC in promoters is negatively associated with gene expression (**Figure** **4**A). However, contrary to previous studies that suggest 5mC in gene bodies correlates with gene activation,^[^ ^46^ ^]^ we found that 5mC was also negatively correlated with gene expression in these regions. In contrast, 5hmC showed a positive correlation with gene expression, regardless of its location in promoters or gene bodies (Figure 4B).

Interestingly, the combined levels of 5mC and 5hmC did not correlate with gene expression in promoters but did show a positive correlation in gene bodies (Figure 4C). This observation may help explain inconsistencies in earlier studies that reported a positive correlation between gene body 5mC and gene expression, as many of these studies did not distinguish between 5mC and 5hmC. This underscores the critical importance of directly identifying 5mC to more accurately understand its role in gene regulation.

Although 5hmC is highly correlated with gene expression, most gene expression changes in AD mice were not associated with alterations in 5hmC or 5mC levels (Figure 4D–H; Figure S8A–H, Supporting Information). This is expected, as gene expression changes can also be influenced by upstream regulatory pathways beyond epigenetic modifications. Nonetheless, we identified specific genes whose expression was affected by changes in 5mC or 5hmC (Figure 4G,H; Figure S8A–D, Supporting Information). Notably, DNA hypomethylation in both gene bodies and promoters in AD led to the upregulation of certain genes, rather than their downregulation, reinforcing the central role of DNA methylation in suppressing gene expression (Figure S8A,C,E, Supporting Information).

Although 5hmC was positively correlated with gene expression, more hypo‐DhMGs were upregulated than downregulated (Figure S8B,D,F, Supporting Information). This suggests that reduced levels of 5hmC are not associated with global gene expression changes in AD, but rather may be linked to other regulatory mechanisms contributing to AD pathogenesis. Nevertheless, several representative genes, such as *Enc1* and *Kif5a*,^[^ ^47^, ^48^ ^]^ lost DNA hydroxymethylation, and their expression was compromised, indicating that a causal relationship between 5hmC and gene expression cannot be ruled out (Figure 4I,J; Figure S8G,H, Supporting Information).

### 2.6 Establishment of CT‐seq as a Validation Method for 5mC Distribution

**Figure 5:**
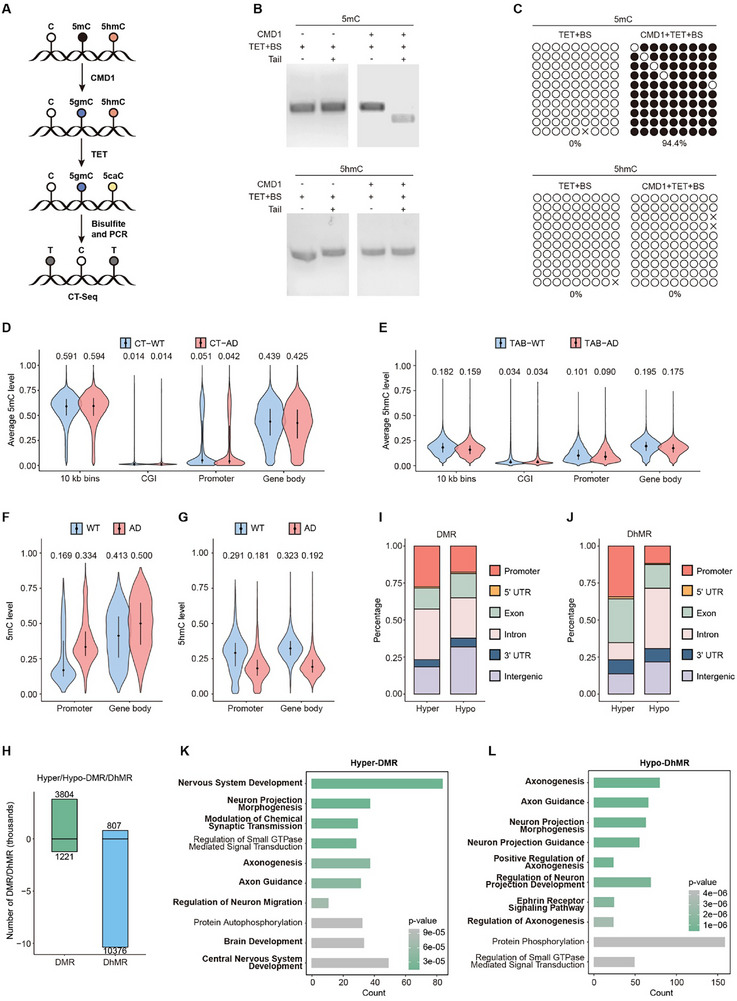
Independent analysis of 5mC and 5hmC by CT‐seq and TAB‐seq validates global changes in DNA modification in AD. A) Schematic diagram of the CT‐seq workflow for 5mC sequencing. B) Restriction endonuclease digestion assay of cytosine modification variants in ACGT contexts following sequential CMD1 and TET enzyme reactions, bisulfite treatment, and PCR amplification. Representative images from two independent biological replicates are shown. C) CT‐seq analysis of 5mC‐ or 5hmC‐DNA. Each circle represents a CpG site in the DNA sequence, with unfilled circles indicating converted cytosines (5mC without CMD1 treatment or 5hmC) and filled black circles representing unaltered ones (5mC with CMD1 treatment). The data shown are representative of two independent biological replicates. The 5mC‐ and 5hmC‐DNA used in these assays were prepared by PCR amplification, and the sequence is provided in the methods section. D,E) Average modification levels of 5mC (D) detected by CT‐seq and 5hmC (E) detected by TAB‐seq in different regions of WT (blue) and AD (red) samples, based on all detected CpG sites. The region ±1 kb from the transcription start site is defined as the promoter. The width of the violin plot represents the frequency of data at each value. The short horizontal line denotes the interquartile range of the data, while the black dot indicates the median. Specific numerical values are annotated for clarity. All the sequencing data were analyzed based on two independent biological replicates, which are summarized in Figure S9A. F,G) Average 5mC levels of DMR (F) and 5hmC levels of DhMR (G) in different regions for WT (blue) and AD (red) samples. DMR/DhMR are defined as regions spanning ≥ 10 CpGs, with a mean modification difference between groups ≥ 0.1 and a MWU‐test *p* value < 0.001. The width of the violin plot represents the frequency of data at each value. The short horizontal line denotes the interquartile range of the data, while the black dot indicates the median. Specific numerical values are annotated for clarity. H) Number of hyper‐DMR, hypo‐DMR, hyper‐DhMR, and hypo‐DhMR. Regions with increased modification levels in AD are classified as hyper‐DMR/DhMR, and those with reduced modification levels as hypo‐DMR/DhMR. I,J) Distribution of hyper/hypo‐DMR (I) and hyper/hypo‐DhMR (J) across different genomic contexts. K,L) Gene Ontology analysis of hyper‐DMR detected by CT‐seq (K) and hypo‐DhMR detected by TAB‐seq (L). The color of each bar represents the *p*‐value, with darker shades indicating greater statistical significance.

While CD‐seq and ACE‐seq leverage DNA deaminases to convert 5hmC or 5mC into T after sequencing, TAB‐seq utilizes TET‐mediated oxidation followed by bisulfite conversion for the detection of 5hmC.^[^ ^37^ ^]^ Following CMD1‐mediated conversion of 5mC into 5gmC, 5hmC is also converted into T through TET oxidation and bisulfite treatment, allowing for the identification of 5mC via CT‐seq (**Figure** **5**A). As a result, CT‐seq offers the distinct advantage of directly differentiating between 5mC and 5hmC (Figure 5B,C), and was used alongside TAB‐seq to validate the distribution of 5mC and 5hmC in WT and AD model mice.

Despite the high utility of these methods, CT‐seq and TAB‐seq exhibited lower conversion efficiencies compared to CD‐seq and ACE‐seq (Figure S9A, Supporting Information), which led to a higher incidence of false positives and fewer confidently detected sites. This discrepancy is likely due to the less efficient activity of the TET enzymes we used in these methods. Nonetheless, CT‐seq successfully revealed distribution patterns consistent with those observed by CD‐seq, with the majority of 5mC detected at CpG sites (Figure S9B, Supporting Information). In contrast, TAB‐seq identified more 5hmC sites in non‐CpG regions, which may be attributed to the relatively low abundance of 5hmC and the higher false‐positive rate associated with this technique (Figure S9C, Supporting Information). Despite these limitations, both CT‐seq and TAB‐seq yielded reliable results across biological replicates, and the distribution patterns were consistent across samples (Figure S9D–I, Supporting Information). The consistency across various genomic regions between CD‐seq and CT‐seq was analyzed, confirming the effectiveness of both methods in 5mC profiling (Figure S10A–D, Supporting Information).

Detailly, we quantified the levels of 5mC and 5hmC across various genomic contexts (Figure S10E,F, Supporting Information). As expected, 5hmC levels were significantly reduced in AD mice, while 5mC levels remained largely unaltered (Figure 5D,E). 5hmC was substantially decreased in DhMR, while 5mC was increased in DMR (Figure 5F–H). Moreover, hyper‐DMR and hypo‐DhMR were more preferentially located in gene body regions (Figure 5I,J). CT‐seq and TAB‐seq also revealed a complex relationship between 5mC and 5hmC, with 5hmC levels being positively correlated with 5mC in promoters but negatively correlated in gene bodies (Figure S10G,H, Supporting Information). This finding further supports the distinct and context‐dependent roles of these DNA modifications. Notably, hypo‐DhMR and hyper‐DMR were enriched in axonogenesis‐related pathways, emphasizing the critical role of 5hmC loss and 5mC gain in AD pathogenesis (Figure 5K,L; Figure S10I,J, Supporting Information).

Finally, the DMC and DhMC identified by CT‐seq and TAB‐seq corroborated the independent changes of these modifications (Figure S11A, Supporting Information). The observed increase of 5mC and decrease of 5hmC in distinct loci suggest that these two modifications play independent regulatory roles in the pathogenesis of AD (Figure S11B–E, Supporting Information).

## 3 Discussion

The independent identification of 5mC and 5hmC is crucial for understanding their distinct roles in development and disease. While several methods have been developed to map the distribution of either 5mC or 5hmC,^[^ ^49^, ^50^ ^]^ a parallel, subtraction‐free approach for simultaneously determining the distribution and abundance of both modifications has been lacking. In this study, we took advantage of CMD1, a TET‐homologue capable of converting 5mC into 5gmC,^[^ ^40^ ^]^ to develop two orthogonal methods for 5mC sequencing: CD‐seq and CT‐seq. These methods, when paired with ACE‐seq or TAB‐seq, allowed us to generate a comprehensive landscape of 5mC and 5hmC in both WT and AD model mice. Our results revealed a substantial reduction of 5hmC levels across the hippocampal genome in AD mice, while 5mC remained largely unchanged. Although 5mC also showed changes at specific loci, the DMC and DhMC sites had minimal overlap, supporting the notion that 5hmC serves as an independent epigenetic mark in the brain.

Compared to TAPSβ, CD‐seq and CT‐seq demonstrate comparable performance in identifying 5mC at single‐base resolution. Notably, CD‐seq shares methodological features with ACE‐seq, while CT‐seq aligns with TAB‐seq, enabling their paired application for the independent and high‐resolution detection of 5mC and 5hmC.

While previous studies have shown a positive correlation between gene body 5mC and gene expression,^[^ ^46^ ^]^ our data demonstrate that 5mC often functions as a repressive mark, regardless of its genomic location. In contrast, 5hmC is consistently associated with active gene expression. These findings underscore the utility of methods like CD‐seq and ACE‐seq, which allow for independent detection of 5mC and 5hmC at single‐base resolution, providing a more precise and detailed view of the DNA epigenetic landscape.

In this study, we observed that more genes were upregulated than downregulated in the AD mice, with hypomethylated loci in both promoters and gene bodies correlating with these upregulated genes. These findings further support the role of 5mC as a repressive mark in gene expression. Although 5hmC is globally associated with active gene transcription, the genes exhibiting significantly reduced 5hmC levels in AD mice, which is strongly linked to neuronal dysfunction, show minimal overlap with differentially expressed genes. This suggests that 5hmC reduction in this pathological context does not directly lead to widespread transcriptional changes at the level of individual affected loci. This points to a more complex role for 5hmC in the brain, where it may influence neuronal function and disease processes through mechanisms not directly tied to transcriptional regulation.

We tried to examine the relationship between 5hmC loci and chromatin accessibility using previously published ATAC‐seq datasets, generated from the hippocampus of APP/PS1 AD mice.^[^ ^51^ ^]^ As previously reported,^[^ ^23^ ^]^ 5hmC enrichment was positively associated with open chromatin regions (Figure S12A, Supporting Information). Consistently, the reduction of 5hmC in the AD mice corresponds to substantial loss of chromatin accessibility, which might result in the alteration of gene expression observed in AD (Figure S12B–D, Supporting Information). Additionally, the observed reduction in 5hmC could be attributed to the downregulation of the three Tet proteins or reduced enzymatic activities (Figure S12E, Supporting Information). However, the precise mechanisms underlying dysregulated 5hmC‐mediated AD pathogenesis remain unclear and warrant further investigation. Notably, the 5hmC loss and the DhMGs identified in our dataset show substantial overlap with previously reported 5hmC‐altered genes in both mouse and human AD studies, supporting a conserved role for 5hmC in AD pathogenesis between human and mice (Table S1, Supporting Information).^[^ ^31^, ^52^ ^]^ Continued research into these processes could provide deeper insights into DNA epigenetics in the brain and help identify potential therapeutic targets for AD.

In summary, our efforts to independently identify 5mC and 5hmC offer significant advantages in elucidating the distinct roles of these DNA modifications and their relationship with gene transcription. However, certain challenges remain, including suboptimal conversion efficiency and the need to enhance the enzymatic activity of CMD1 and TET, which may contribute to the slight discrepancies observed between CD‐seq and CT‐seq. Additionally, the development of more efficient deaminases capable of distinguishing a broader range of DNA modifications would further refine these methodologies and expand our understanding of epigenetic regulation.

## 4 Conclusion

In this study, we developed two orthogonal methods: CD‐seq and CT‐seq, which, together with previously established ACE‐seq and TAB‐seq, enable the independent sequencing of 5mC and 5hmC, thereby expanding the epigenetic toolkit. Notably, we found that 5hmC plays a crucial role in AD pathogenesis. Although 5hmC levels are significantly reduced in the AD brain, this change has minimal impact on 5mC levels and subsequent gene expression, suggesting a more complex and independent role for 5hmC in AD. Our findings underscore the importance of independently quantifying each DNA modification and highlight 5hmC as a distinct epigenetic mark in mammalian cells.

## 5 Experimental Section

### CMD1 Protein Expression and Purification

The pPEI‐His‐SUMO‐CMD1 expression plasmid was constructed as previously reported^[^ ^40^ ^]^ and transformed into *E. coli* strain BL21(DE3) (Tsingke). When the absorbance at OD600 reached 0.6, 0.5 mм Isopropyl‐β‐D‐thiogalactoside (IPTG) was added to induce protein expression. The bacterial cells were cultured at 16 °C for 16 h, and then harvested by centrifugation. The cells were lysed under high pressure, and the lysate was centrifuged at 12 000 × g for 40 min. The supernatant was incubated with Ni‐NTA beads (Vazyme) at 4 °C for 4 h. The beads containing the His‐tagged CMD1 protein were washed, then incubated with His‐tagged Ulp1 protease at 4 °C for 19 h to cleave the His‐tag. The purified CMD1 protein was collected and stored in storage buffer (50 mм HEPES pH 7.0, 50 mм NaCl). Finally, the protein was concentrated using Microsep Advance centrifugal devices (PALL).

### Preparation of 5mC and 5hmC Spike‐in Controls

For the 5mC spike‐in control, methylated lambda DNA was used. To generate this, 1 µg of lambda phage DNA (λDNA, dam‐, dcm‐; Takara) was incubated with M.SssI methyltransferase (NEB) at 37 °C for 4 h in the presence of 1× reaction buffer and 160 µM S‐adenosylmethionine (SAM), following the manufacturer's instructions. After methylation, the DNA was treated with proteinase K (NEB) to remove proteins, then purified using the DNA Clean & Concentrator kit (Zymo) according to the manufacturer's protocol.

For the 5hmC spike‐in control, a random 480‐bp template DNA was synthesized with the following sequence: 5’‐GAATTCTTGCAGCACTAGTGCATCTATAAGTTATCTCAAATCAAGAAATCAGTCTAATGAGAATTTCAATAACTTCAGCAATTTAAGCTGCATGCATCAGTGTCATCGTTATTTTTTTTTTGAGACGTAGTCATGCTCTGTTGCTGAGTCTGCAGTACAGTGACGAGATATCGACTCAGCACAACATCTGCATCACATGTTCAAGCGATTCTCATGCTTCAGCTTGCAGAGTAGCTGTCACTACAGACACTGAGCAGCATGCGTGACTAATTTTTGTATTTTTAGTAGAGAGTGCATTTCGTCATGTTGTACAGTCTAGTTTCAAACTCATGACTTCAGTTGATCTAACTGACACGATCTCAGAATTTACTGTCATTACAGTACTGTCACACAGTGACAGTCATTTTTCTTAATTTTTAAAAATATTAAAGTTTTATCTCATTCGTGTTGAAGCATATTCGTGATTTAAAAGTTGCAAAG‐3’.

The PCR amplification was performed using TaKaRa Taq^TM^ Hot Start Version (TaKaRa) with the following primers: Forward primer: GAATTCTTGCAGCACTAGTGCATCTC; and Reverse primer: CTTTGCAACTTTTAAATCAC. In this reaction, 5‐hydroxymethyl‐dCTP (5hm‐dCTP) was used instead of the unmodified dCTP, ensuring that the resulting PCR product was fully hydroxymethylated at cytosines. The amplified DNA was then purified using the SanPrep Column DNA Gel Extraction Kit (Sangon) according to the manufacturer's instructions. Notably, the 5hmC spike‐in sequence was also used to prepare 5mC‐DNA, which was amplified by PCR and used in the CMD1 reaction in Figure 1C,D, and Figure 5B,C.

### CMD1, TET, T4‐βGT and DEA Activity Assay

For the CMD1 reaction, 100 ng of DNA was treated with 5 µg of CMD1 protein in a 100 µl reaction system containing the reaction buffer (50 mм HEPES pH 7.0, 50 mм NaCl, 3 mм ascorbic acid, and 0.25 mм Fe(NH_4_)_2_(SO_4_)_2_). The reaction was incubated at 37 °C for 2 h.

For the TET or T4‐βGT reaction, 100 ng of DNA was treated with TET (Vazyme, EM301‐C104) or T4‐βGT (NEB) in the presence of the respective reaction buffer and incubated at 37 °C for 2 h, following the manufacturer's instructions.

For the DEA reaction, 100 ng of DNA was first denatured to single strands by incubating with 0.1 M NaOH at 50 °C for 10 min, followed by immediate chilling on ice. Then, 1 µl of DEA deaminase (Vazyme, EM301‐C110) was added along with 10 µl of DEA reaction buffer and 1 µl of BSA. The reaction was carried out at 37 °C for 4 h.

After the reaction, the treated DNA was incubated with proteinase K (NEB) and further purified using the DNA Clean & Concentrator‐5 Kit (Zymo Research) according to the manufacturer's instructions.

### ES Cell Culture

Mouse embryonic stem (ES) cells were maintained at 37 °C with 5% CO_2_ in DMEM (high glucose with Ala‐Gln, MeilunCell) supplemented with 10% Fetal Bovine Serum (FBS, MeilunCell), 0.1 mм β‐mercaptoethanol, 1× MEM non‐essential amino acids (BasalMedia), 1× EmbryoMax Nucleosides (Sigma–Aldrich), 1000 units Leukemia Inhibitory Factor (LIF, MeilunCell), 3 mм Laduviglusib (CHIR99021, Selleck), and 1 mм Mirdametinib (PD0325901, Selleck). The cell culture dishes were pre‐treated with 0.1% gelatin solution (Beyotime) to promote adhesion.

### Mouse Breeding

The mice used in this study were obtained from Jackson Laboratory and maintained under pathogen‐free conditions. All experiments were conducted in accordance with the guidelines of Tongji University for the use of animals in biological research. The experimental protocol was approved by the Tongji University Animal Care and Use Committee, Shanghai, China, on August 25, 2020 (approval number TJAB04120102).

### Genomic DNA and RNA Extraction

Tissues, including the cerebellum, cerebral cortex, hippocampus, liver, kidney, spleen, and muscle, were dissected from both WT and AD mice. These tissue samples were homogenized and digested with proteinase K at 55 °C for 1 h. For cultured ES cells, the cells were harvested and washed with PBS. Genomic DNA was extracted from both tissues and cells using the Wizard Genomic DNA Purification Kit (Promega) following the manufacturer's instructions.

Total RNA from the hippocampus was extracted using Trizol reagent (Sangon) according to the manufacturer's instructions.

### Quantification of DNA Modifications by UPLC‐MS/MS Analysis

To quantitatively determine the content of each nucleoside, genomic DNA and enzyme‐treated DNA fragments were first digested by nuclease P1 (NEB) at 37 °C for 1 h, followed by dephosphorylation with Quick CIP (NEB) at 37 °C for an additional 1 h. The samples were then centrifuged, and the supernatants were collected for MS analysis. DNA modifications were quantified using an ACQUITY UPLC system (Waters) coupled to a Triple Quad^TM^ 6500+ LC‐MS/MS system (SCIEX) in multiple reaction monitoring (MRM) mode. Separation was performed on an ACQUITY Premier HSS T3 column (100 Å, 1.8 µm, 2.1 × 100 mm, Waters) with the following mobile phases: A (water with 0.1% formic acid) and B (100% methanol), at a flow rate of 0.3 mL min^−1^. The linear gradient started with 100–95% A (0–2 min), followed by 95‐90% A (2–4 min), 90‐50% A (4–7 min), 50–5% A (7–7.5 min), 5% A (7.5–9 min), 5–100% A (9–9.1 min), and 100% A (9.1–10 min). Optimized MRM transition parameters for each nucleoside were determined using pure compound standards. The quantifier transitions for each nucleoside were as follows: 5mC: 242.1/126.1 (CE 17, DP 20); 5hmC: 258.1/142.1 (CE 15, DP 40); dC: 228.1/112.1 (CE 20, DP 20); dG: 268.1/152.1 (CE 20, DP 60); 5gmC: 332.1/216.1 (CE 20, DP 15); 5fC: 256.1/140.1 (CE 16, DP 30); 5caC: 272.1/156.1 (CE 15, DP 20). All compounds were measured in positive ESI mode. Retention times for each nucleoside on the HSS T3 column were observed as follows: 5mC: 3.15 min; 5hmC: 2.24 min; dC: 2.31 min; dG: 4.50 min; 5gmC: 2.71 min; 5fC: 4.67 min; 5caC: 3.11 min. The amount of each nucleoside was quantified by interpolating the peak areas of the quantifier MRM transitions from the standard curves.

### ACE‐Seq and TAB‐Seq

For both ACE‐seq and TAB‐seq, 100 ng of genomic DNA containing 0.5% 5mC spike‐in and 0.5% 5hmC spike‐in was used.

For ACE‐seq,^[^ ^38^ ^]^ DNA containing the spike‐in was sheared to ≈300 bp using a Covaris M220. T4‐βGT (NEB) was added to convert 5hmC into 5ghmC according to the manufacturer's instructions, and the reaction mixture was incubated at 37 °C for 2 h. The DNA was purified using the DNA Clean & Concentrator‐5 Kit (Zymo Research) according to the manufacturer's instructions. The purified DNA was then denatured by treatment with 0.1 m NaOH at 50 °C for 10 min, followed by deamination using the DEA enzyme mix (Vazyme) at 37 °C for 4 h, according to the manufacturer's protocol.

For TAB‐seq,^[^ ^37^ ^]^ DNA containing the spike‐in was sheared to ≈600 bp using the Covaris M220. T4‐βGT (NEB) was added with related buffer following the manufacturer's instructions, and the reaction mixture was incubated at 37 °C for 2 h. The DNA was purified using the DNA Clean & Concentrator‐5 Kit (Zymo Research) according to the manufacturer's instructions. The purified DNA was then incubated with TET proteins (Vazyme) at 37 °C for 2 h in the presence of the related buffer, following the manufacturer's instructions, and then bisulfite conversion was performed using the EZ DNA Methylation‐Direct kit (Zymo), according to the manufacturer's protocol.

After the reactions, DNA was purified and quantified using a Qubit fluorometer (Thermo). Library preparation was performed using the EpiArt DNA Methylation Library Kit for Illumina V3 (Vazyme), following the manufacturer's instructions. Libraries were analyzed using an Agilent 2100 Bioanalyzer and sequenced on an Illumina NovaSeq X Plus platform with 150 bp paired‐end sequencing.

### CD‐Seq and CT‐Seq

For both CD‐seq and CT‐seq, 500 ng of genomic DNA containing 0.5% 5mC spike‐in and 0.5% 5hmC spike‐in was used.

For CD‐seq, DNA was incubated with CMD1 protein to convert 5mC into 5gmC as described above. After purification using the DNA Clean & Concentrator‐5 Kit (Zymo Research), the reaction was repeated once to improve the conversion efficiency. The DNA was then sheared to ∼300 bp using a Covaris M220. The DNA was concentrated and denatured by treatment with 0.1 M NaOH at 50 °C for 10 min, followed by deamination with the DEA enzyme mix (Vazyme) at 37 °C for 4 h, according to the manufacturer's protocol.

For CT‐seq, DNA was incubated with CMD1 protein to convert 5mC into 5gmC as described above. After purification using the DNA Clean & Concentrator‐5 Kit (Zymo Research), the reaction was repeated once to improve the conversion efficiency. The DNA was then sheared to ∼600 bp using a Covaris M220. The DNA was concentrated and treated with TET proteins (Vazyme) at 37 °C for 2 h in the presence of the appropriate buffer, following the manufacturer's instructions. Bisulfite conversion was performed using the EZ DNA Methylation‐Direct kit (Zymo Research), according to the manufacturer's protocol.

Library preparation and sequencing were performed as described above for ACE‐seq and TAB‐seq.

### RNA Sequencing

RNA‐seq transcriptome libraries were prepared following the Illumina® Stranded mRNA Prep, Ligation protocol using 1 µg of total RNA. Briefly, messenger RNA was isolated using the polyA selection method with oligo(dT) beads, followed by fragmentation with a fragmentation buffer. Next, double‐stranded cDNA was synthesized using the SuperScript Double‐Stranded cDNA Synthesis Kit (Invitrogen) with random hexamer primers (Illumina). The synthesized cDNA was then subjected to end‐repair, phosphorylation, and ‘A’ base addition according to Illumina's library construction protocol. Libraries were size‐selected for cDNA target fragments of 300 bp on a 2% Low Range Ultra Agarose gel, followed by PCR amplification using Phusion DNA Polymerase (NEB) for 15 cycles. After quantification with the Qubit 4.0, the paired‐end RNA‐seq libraries were sequenced using the NovaSeq X Plus sequencer (Illumina).

### Data Preprocessing of CD‐Seq, CT‐Seq, ACE‐Seq and TAB‐Seq

Sequencing reads were trimmed using Trim Galore! v0.6.10 (https://github.com/FelixKrueger/TrimGalore) to remove adapters and low‐quality bases. The trimmed reads were then mapped to a combined genome of spike‐in sequences and the mm39 mouse genome using BASAL^[^ ^53^ ^]^ v1.8. PCR duplicates were removed using the markdup function of Sambamba^[^ ^54^ ^]^ v1.0.1. Modified bases were called using the avgmod function of BASALkit,^[^ ^53^ ^]^ a downstream analysis toolkit for BASAL. Raw signal at each C site was calculated as the ratio of C bases to the sum of C + T bases.

### Statistical Calling of 5mC and 5hmC

A binomial distribution model was used to calculate the significance of methylation or hydroxymethylation. The probability 𝑝 of the binomial distribution was the false‐positive rate of CD‐seq, CT‐seq, ACE‐seq, or TAB‐seq, calculated from the spiked‐in DNAs. Only sites with a Benjamini‐Hochberg adjusted *p* value ≤ 0.05 and read coverage ≥ 5 were considered methylated or hydroxymethylated.

### Data Preprocessing of RNA‐Seq

Sequencing reads were trimmed using Trim Galore! v0.6.10. The trimmed reads were aligned to the mm39 genome using STAR^[^ ^55^ ^]^ v2.7.10b with default parameters. Gene expression counts were generated with featureCounts^[^ ^56^ ^]^ v2.0.6, and differential gene expression was calculated using DESeq2^[^ ^57^ ^]^ v1.44.0. Genes with a *p* value < 0.05 and a log2 fold‐change > 0.58 or < ‐0.58 were considered differentially expressed.

### Identification of DMC, DhMC, DMR, and DhMR

Statistical tests were performed at each CpG site to identify differentially methylated cytosines (DMC) and differentially hydroxymethylated cytosines (DhMC) between the WT and AD groups using the DSS^[^ ^58^ ^]^ v2.52.0 package in R (v4.4.1). DMC and DhMC with a *p* value < 0.05 and a mean modification difference ≥ 0.1 were considered significantly different. Differentially methylated regions (DMR) and differentially hydroxymethylated regions (DhMR) were identified using Metilene^[^ ^59^ ^]^ v0.2‐8 with the following criteria: at least 10 differentially methylated CpGs, an absolute average modification ratio difference ≥ 0.1, a distance between CpGs < 300 bp, and a MWU‐test *p* value < 0.001. Only CpG sites with coverage ≥ 5 were included in the analysis of DMC, DhMC, DMR, and DhMR. Additionally, DMG (Differential methylation genes) and DhMG (Differential hydroxymethylated genes) were defined as genes containing at least one DMR or DhMR within their promoters (± 1 kb) or gene body regions. The list of DhMG was provided in Table S1 (Supporting Information).

### Statistics

Data were presented as mean ± SD. Statistical significance was assessed with two‐way ANOVA. A *p*‐value < 0.05 was considered significant. Statistical analysis was carried out using GraphPad Prism 9 and R (v4.4.1).

### Ethics Approval Statement

No human subjects were involved in this study. The animal ethical and care guidelines were approved by the Tongji University Animal Care and Use Committee, Shanghai, China, on August 25, 2020 (approval number TJAB04120102).

## Conflict of Interest

The authors declare no conflict of interest.

## Author Contributions

Z.W., F.C., and B.H. contributed equally to this work. J.X. conceived the project. Z.W., B.H., W.M., W.Z., Y.F., S.S., Z.S., H.J., and K.Z. performed experiments. F.C. and F.W. analyzed the sequencing data. J.C. and Y.S. bred the mice and collected the tissue samples. Y.H., J.S., and J.X. supervised the study. Z.W., Y.H., J.S., and J.X. wrote the paper. All authors discussed the results and edited the paper.

## Supporting information



Supporting Information

Supplemental Table S1

## Data Availability

The data that support the findings of this study are available on request from the corresponding authors. Public sequencing data used in this study were obtained from the Gene Expression Omnibus (GEO) under the accession number GSM4708553 and GSE145906. Raw data for all sequencing experiments have been deposited at GEO with the accession number GSE290949, GSE290950 and GSE290951.
