# Supplementary material for: Direct Sequencing of 5‐Methylcytosine and 5‐Hydroxymethylcytosine at Single‐Base Resolution Unravels Their Distinct Roles in Alzheimer's Disease
Source: Adv Sci (Weinh). 2025 Jul 16;12(38):e07843. doi: 10.1002/advs.202507843 (PMC12520568; doi:10.1002/advs.202507843)
Supplement: Supplementary file 1 — Supporting Information [file ADVS-12-e07843-s001.docx]

Supporting Information

Direct Sequencing of 5-Methylcytosine and 5-Hydroxymethylcytosine at Single-Base Resolution Unravels Their Distinct Roles in Alzheimer’s Disease

Zi-Xin Wang, Faying Chen, Bao-Dan He, Fan-Chen Wang, Jiaxue Cha, Yu Song, Wei-Ying Meng, Wan-Yue Zou, Yu-Tao Fu, Shu-Xia Sun, Zhi-Yan Sun, Hao-Ming Jiang, Ke-Yao Zhao, Yujun Hou*, Jiejun Shi*, and Jian-Huang Xue*

Supplementary Figures


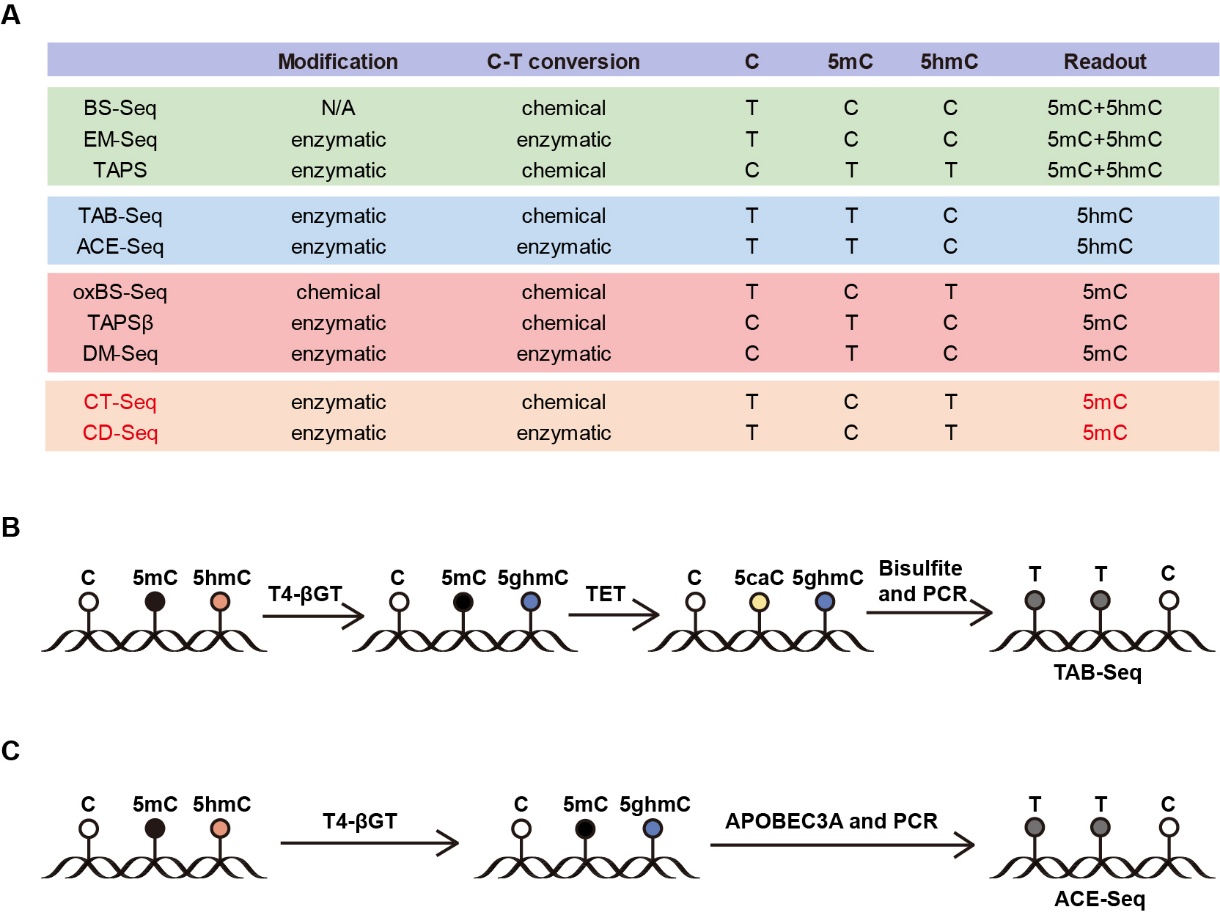


**Figure S1. Comparison of sequencing methods for 5mC and 5hmC.**

**A)** Comparison of various methods for 5mC and 5hmC sequencing.

**B-C)** Schematic diagrams of TAB-seq (**B**) and ACE-seq (**C**). 5mC: 5-methylcytosine; 5hmC: 5-hydroxymethylcytosine; 5caC: 5-carboxylcytosine; 5ghmC: 5-glucosylated-hydroxymethylcytosine; 5gmC: 5-glyceryl-methylcytosine.


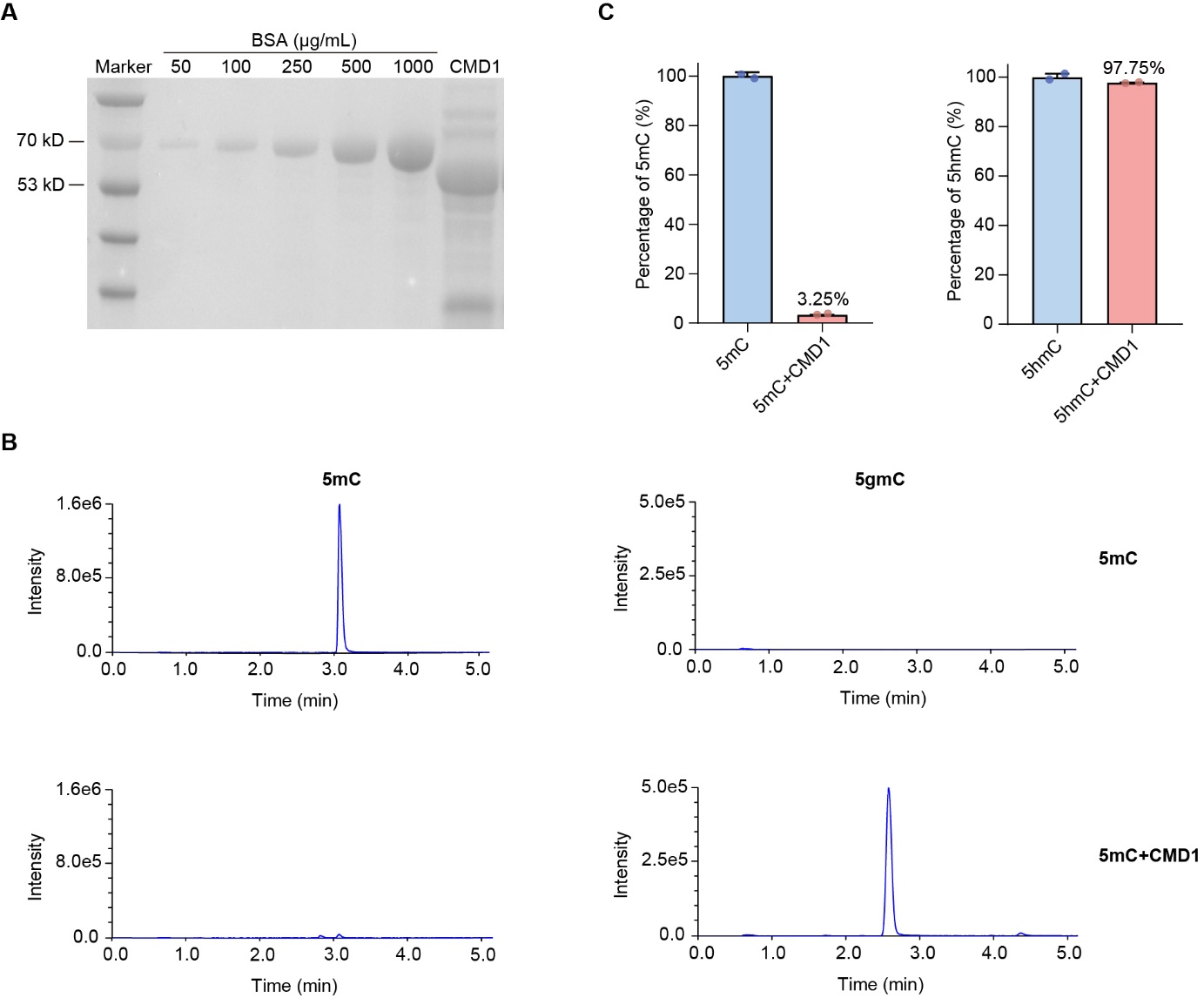


**Figure S2. CMD1 protein purification and reaction assay.**

**A)** Coomassie blue staining of CMD1 proteins purified from *E. coli*.

**B)** Representative image showing mass spectrometry analysis of 5mC and 5gmC before and after CMD1 reaction from two independent technical replicates.

**C)** Relative abundance of 5mC and 5hmC before and after CMD1 treatment. Data are presented as mean ± SD from two independent biological replicates.


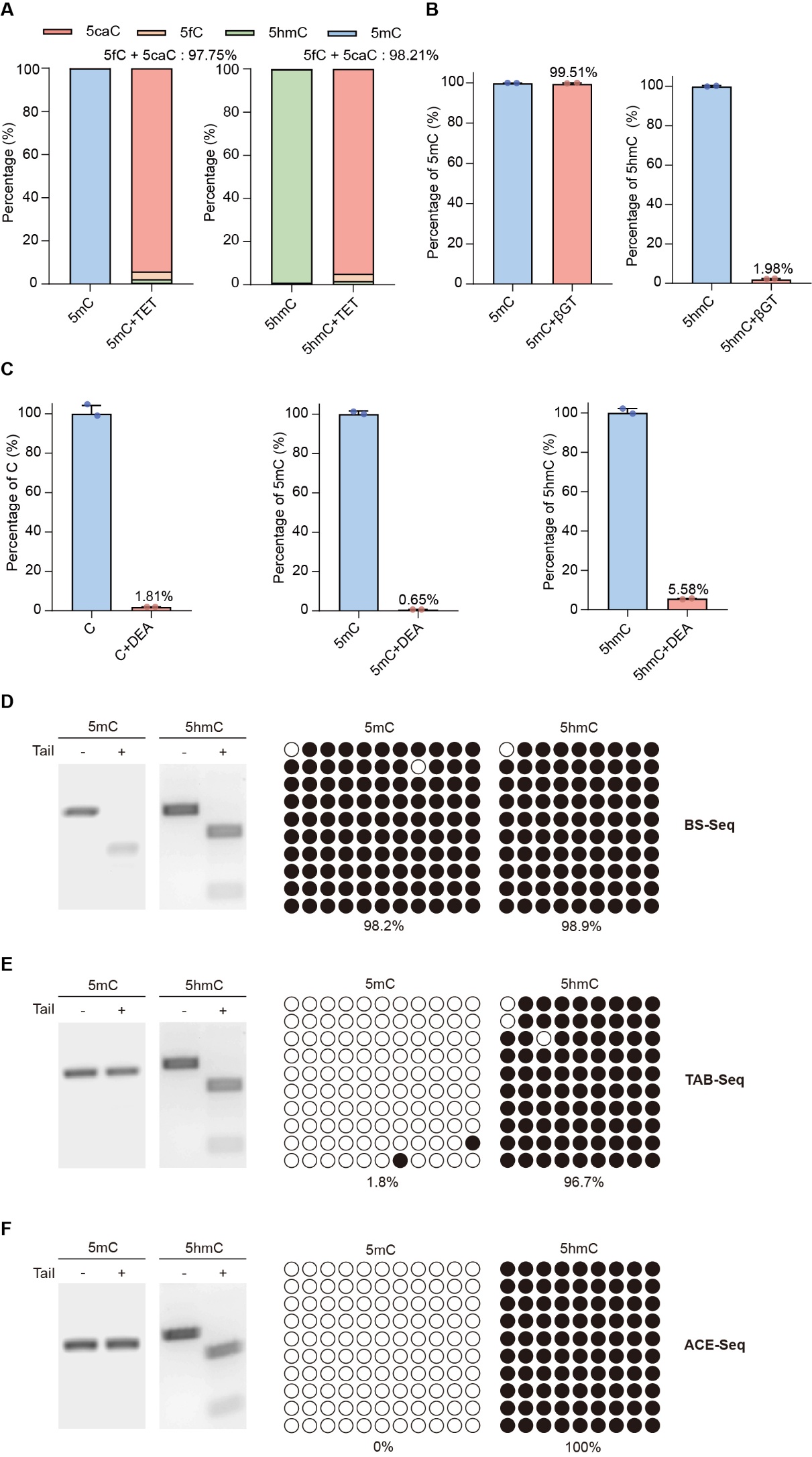


**Figure S3. Validation of TAB-seq and ACE-seq.**

**A-C)** Relative abundance of 5mC and 5hmC after treatment with TET (**A**), βGT (**B**), or DEA (**C**). Data are presented as mean ± SD from two independent biological replicates.

**D-F)** Restriction endonuclease digestion analysis and Sanger sequencing of 5mC and 5hmC using BS-seq (**D**), TAB-seq (**E**), and ACE-seq (**F**). Each circle represents a CpG site in the DNA sequence, with unfilled circles representing converted cytosines (C in BS-seq, 5mC and C in TAB-seq or ACE-seq) and black-filled circles representing unaltered 5mC or 5hmC. All the results presented are based on two biologically independent experiments.


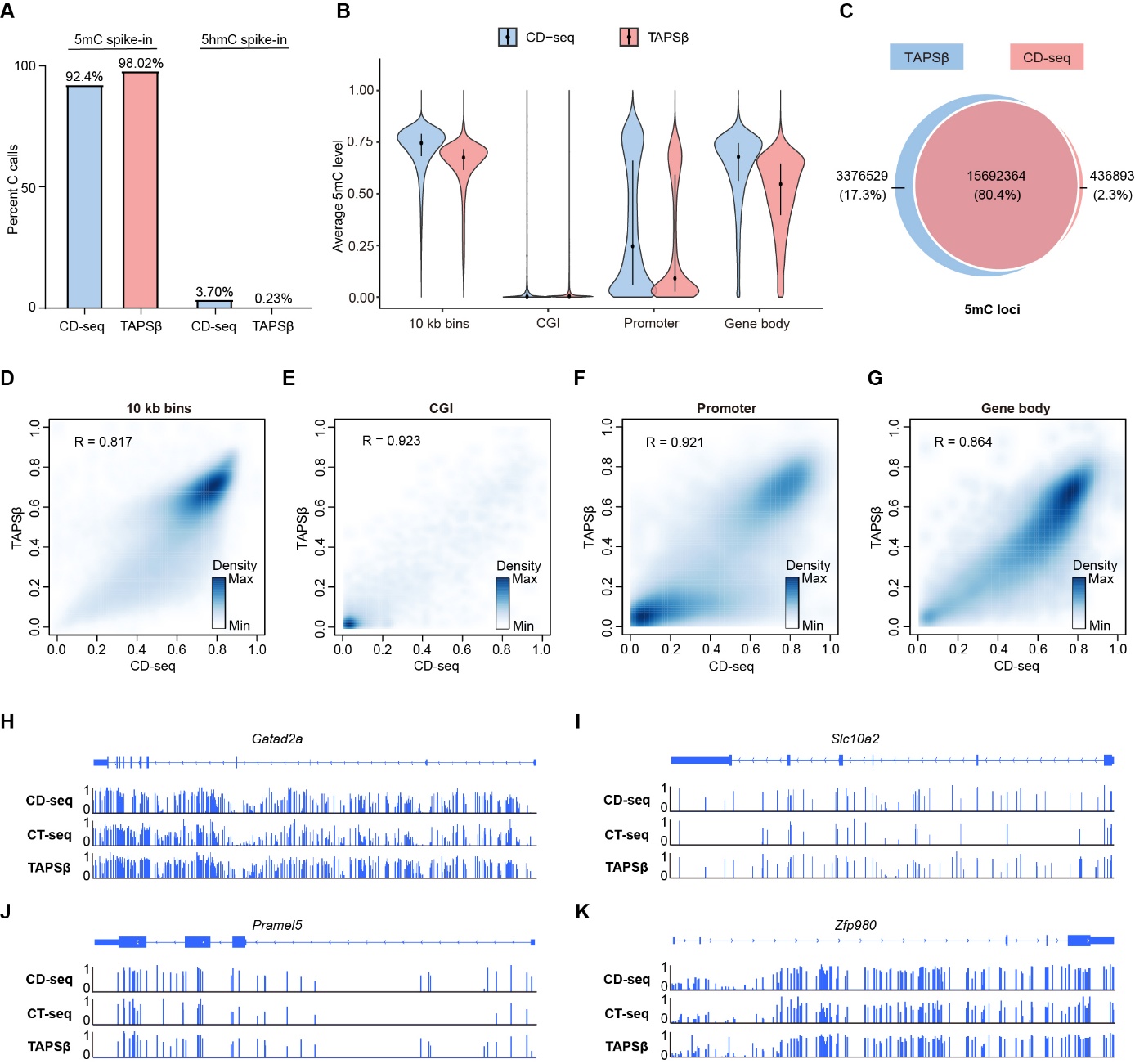


**Figure S4. Comparison of CD-seq and TAPSβ data of genomic DNA from mESCs.**

**A)** Conversion rates of 5mC spike-in and 5hmC spike-in for CD-seq and TAPSβ.

**B)** Average 5mC levels from CD-seq and TAPSβ in different regions. The region ±1 kb from the transcription start site is defined as the promoter. The width of the violin plot represents the frequency of data at each value. The short horizontal line denotes the interquartile range of the data, while the black dot indicates the median.

**C**) Overlap analysis of 5mC loci identified from CD-seq and TAPSβ.

**D-G)** Correlation between CD-seq and TAPSβ at 10 kb bins (**D**), CGIs (**E**), promoters (**F**), and gene bodies (**G**), based on modification levels. Each point represents a 10 kb bin (**D**), a CGI (**E**), a promoter (**F**) or a gene body (**G**), while the color indicating the point density.

**H-K)** Genome browser views showing 5mC distribution at representative genes identified from different methods: *Gatad2a* (**H**), *Slc10a2* (**I**), *Pramel5* (**J**), and *Zfp980* (**K**).


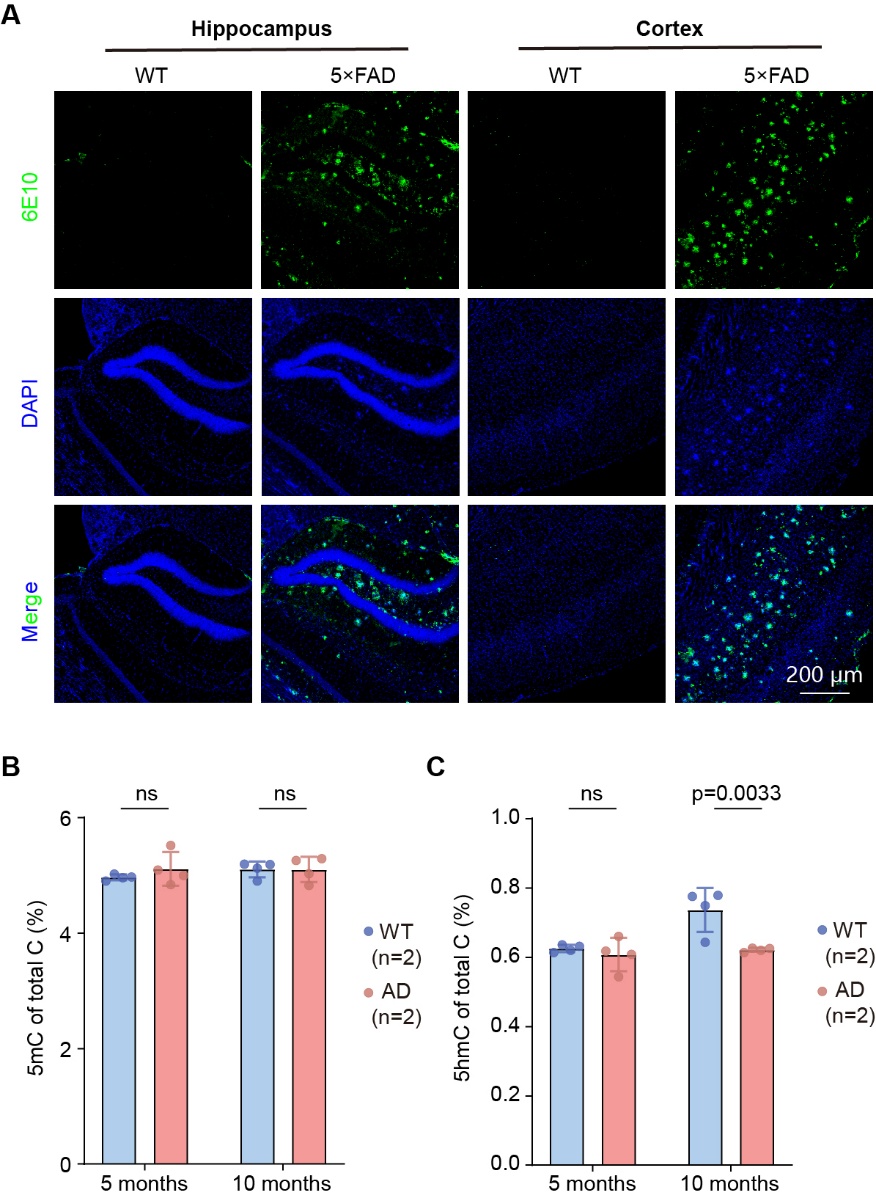


**Figure S5. The degree of differential 5hmC increases progressively with age and disease progression in AD model mice compared to WT controls.**

**A)** Immunofluorescence showing Aβ deposition in the hippocampus and cortex of 15-month-old WT and AD mice. Immunostaining was performed using the 6E10 antibody (targeting Aβ). Representative images are shown from three biological replicates. Scale bar: 200 μm.

**B-C)** MS quantification of 5mC (**B**) or 5hmC (**C**) in the hippocampus of 5- or 10-month-old WT and AD mice. The entire hippocampus was collected for genomic DNA extraction and downstream analyses. Data are presented as mean ± SD from two independent biological replicates. *p* values were determined by two-way ANOVA. Statistical significance was defined as *p* < 0.05; ns indicates not significant.


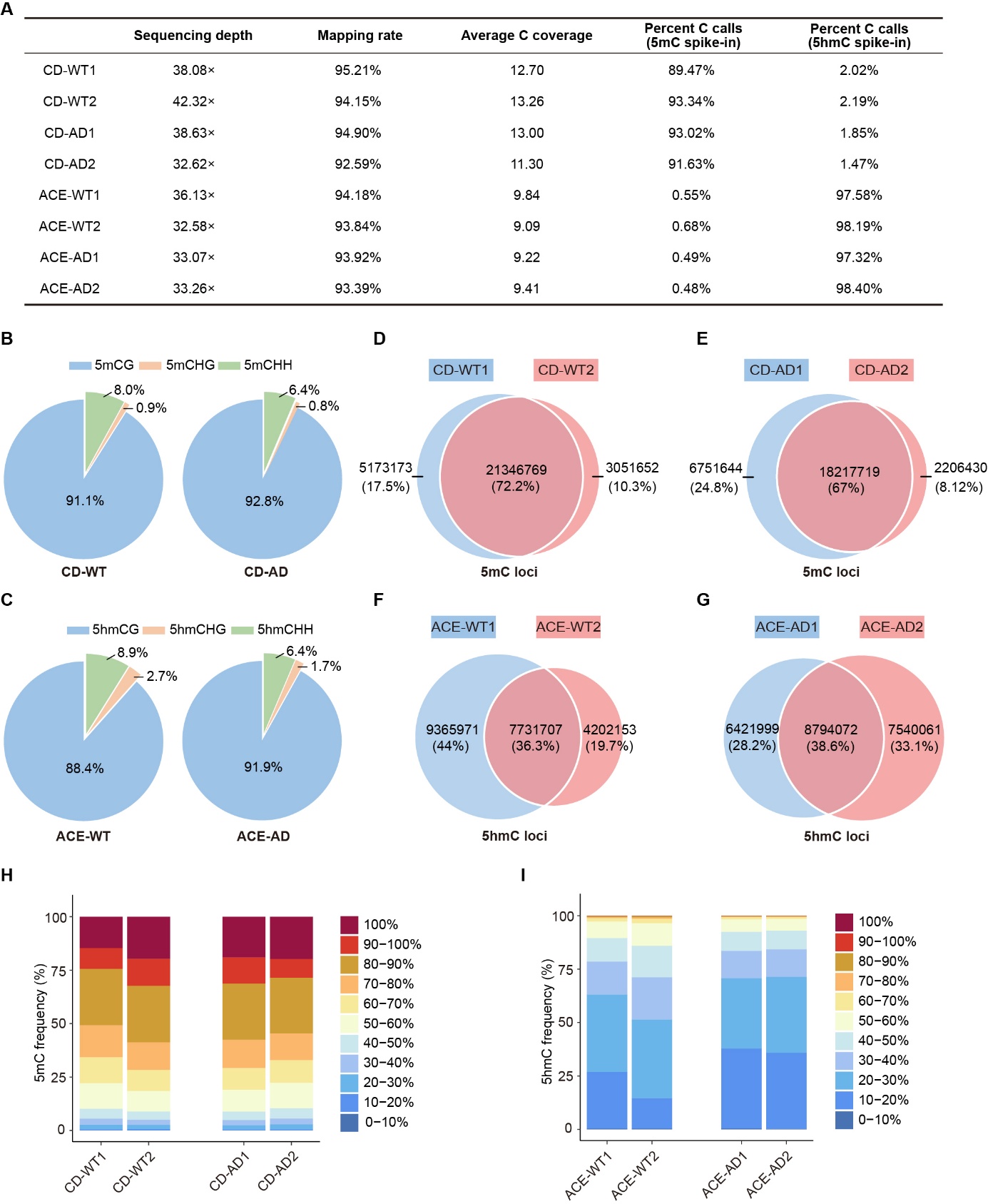


**Figure S6. Genome-wide profiling of 5mC and 5hmC in WT and AD samples using CD-seq and ACE-seq.**

**A)** Summary of filtered sequencing data for each sample using CD-seq and ACE-seq.

**B-C)** Site-specific distribution of 5mC (**B**) and 5hmC (**C**) in CpG, CHG, and CHH contexts in WT and AD samples.

**D-E)** Venn diagrams of overlapping 5mC loci obtained by CD-seq between two biological replicated samples from WT (**D**) and AD (**E**) mice.

**F-G)** Venn diagrams of overlapping 5hmC loci obtained by ACE-seq between two biological replicates of WT (**F**) and AD (**G**) samples.

**H-I)** Frequency distribution of methylation levels determined by CD-seq (**H**) and hydroxymethylation levels determined by ACE-seq (**I**) for each WT and AD sample. The color gradient represents different levels of methylation or hydroxymethylation, ranging from 0-10% (dark blue) to 100% (dark red).


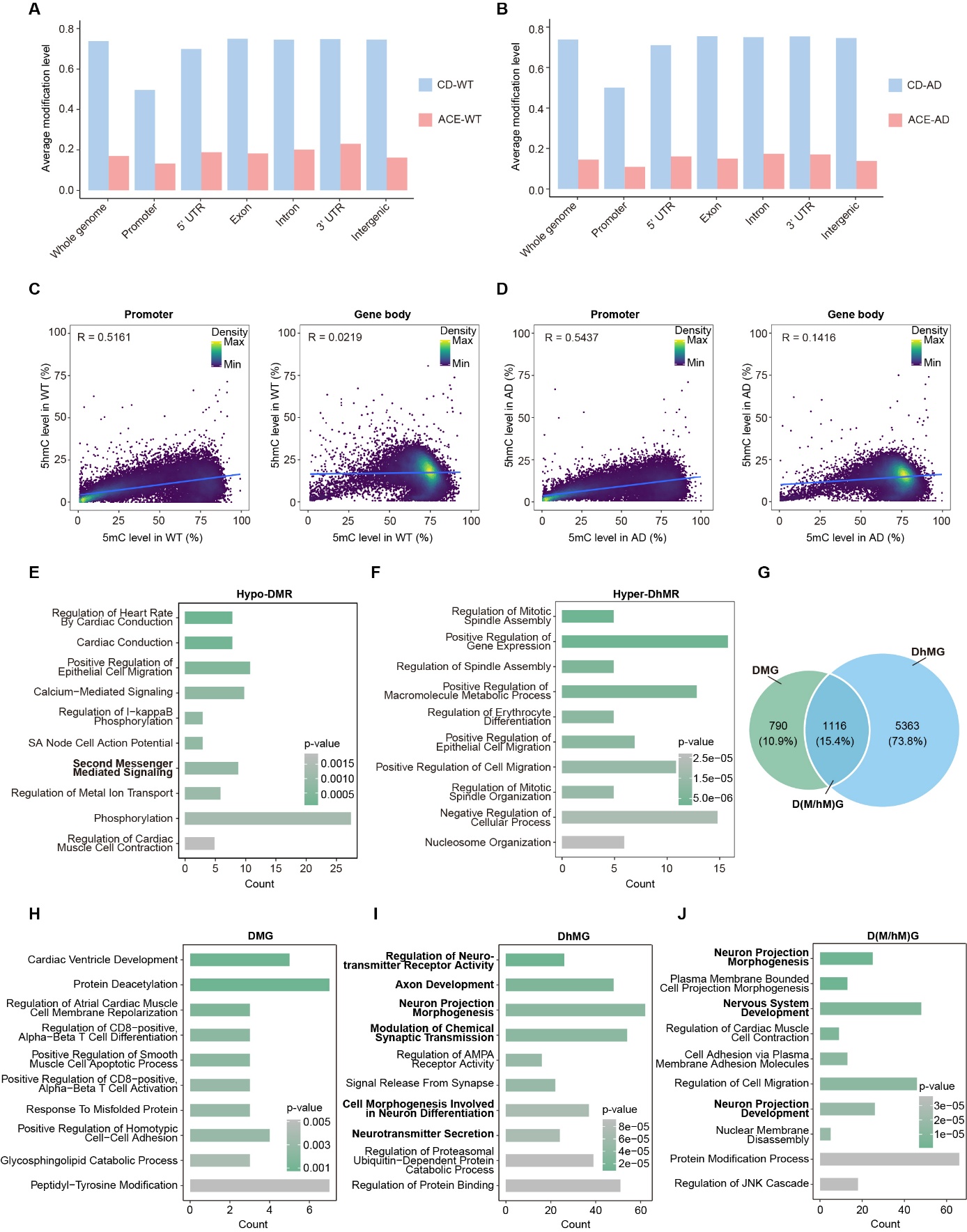


**Figure S7. 5hmC change is correlated with AD pathogenesis.**

**A-B)** Average 5mC (blue) and 5hmC (red) levels in various genomic regions of WT (**A**) and AD (**B**) samples.

**C-D)** Correlation between 5mC and 5hmC levels in promoters (left) and gene bodies (right) of WT (**C**) and AD (**D**) samples. Each point represents a gene, while the color indicating the point density.

**E-F)** Gene ontology analysis of hypo-DMRs from CD-seq (**E**) and hyper-DhMRs from ACE-seq (**F**). The color of each bar represents the *p* value, with darker shades indicating lower *p* values.

**G)** Overlap analysis of DMG and DhMG. Genes in the overlapping region are defined as D(M/hM)G.

**H-J)** Gene ontology analysis of DMG (**H**), DhMG (**I**), and D(M/hM)G (**J**). The color of each bar corresponds to the *p* value, with darker shades indicating lower *p* values.


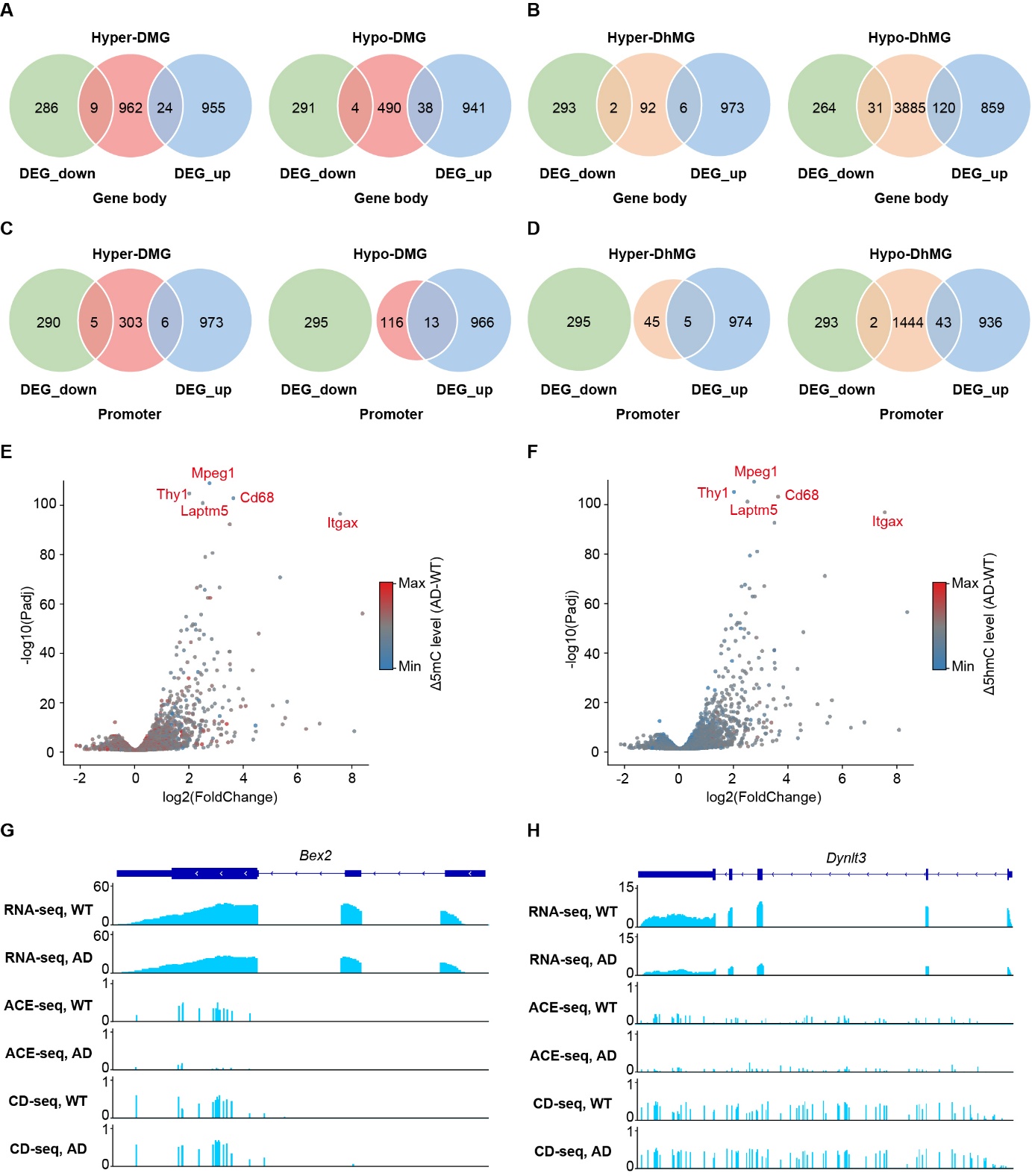


**Figure S8. Global changes in gene expression are not correlated with 5hmC alteration in AD.**

**A-D)** Overlap analysis between DEG and gene body DMG (**A**), gene body DhMG (**B**), promoter DMG (**C**) and promoter DhMG (**D**).

**E-F)** Volcano plot showing gene expression changes versus *p* value for DEG. Genes are color-coded based on their Δ5mC (**E**) level (AD - WT), or Δ5hmC (**F**) levels (AD - WT), with red indicating increased modification in AD compared to WT and blue indicating decreased modification levels. Notable genes with significant expression changes are labeled.

**G-H)** Genome browser views showing expression and methylation/hydroxymethylation levels of representative genes: *Bex2* (brain expressed X-linked 2, chrX:134,965,494-134,971,836) (**G**) and *Dynlt3* (dynein light chain Tctex-type 3, chrX:9,513,174-9,538,545) (**H**). For CD-seq and ACE-seq, each vertical bar represents a CpG site.


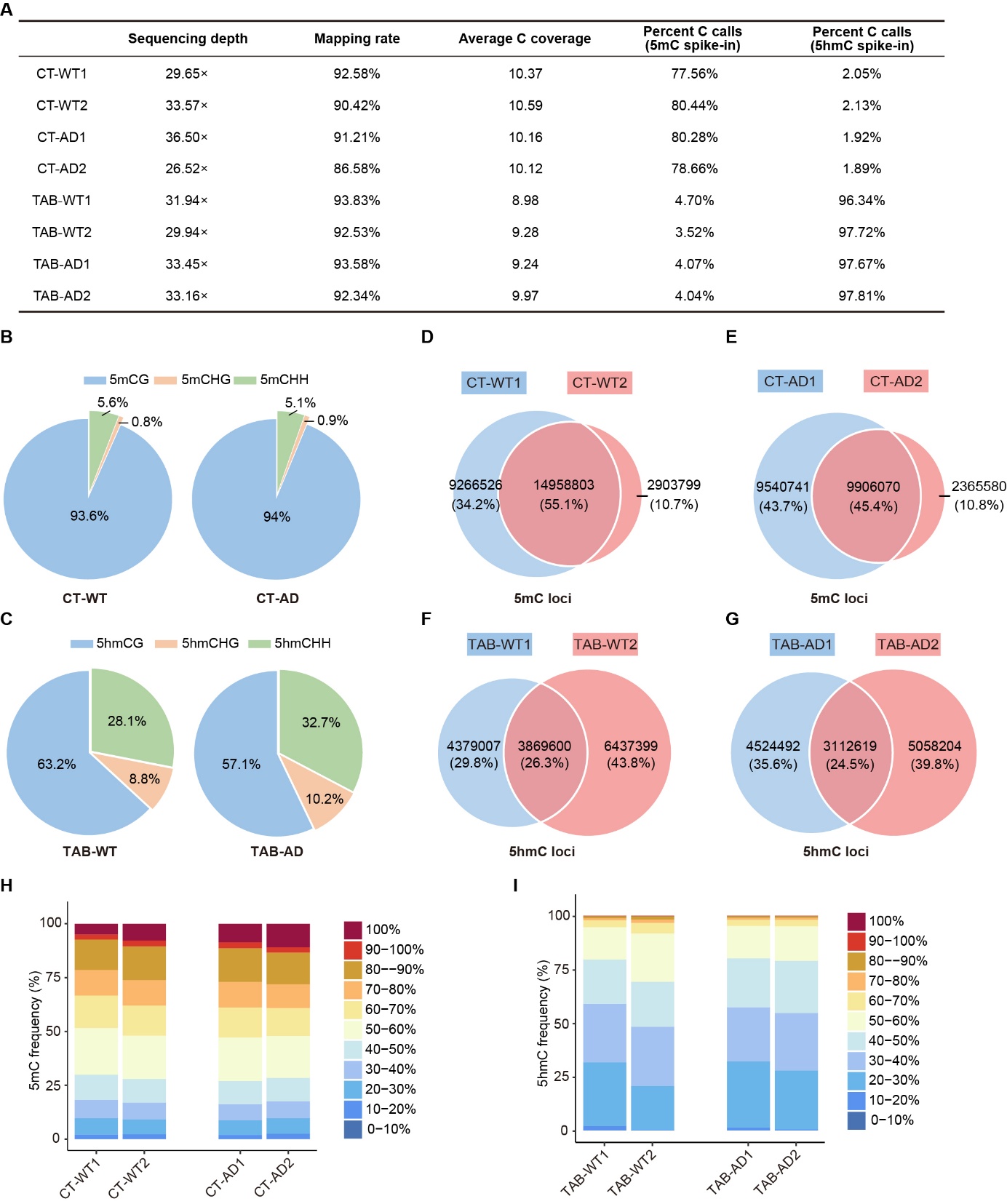


**Figure S9. Genome-wide profiling of 5mC and 5hmC in WT and AD samples using CT-seq and TAB-seq.**

**A)** Summary of filtered sequencing data for each sample using CT-seq and TAB-seq.

**B-C)** Site-specific distribution of 5mC (**B**) and 5hmC (**C**) in CpG, CHG, and CHH contexts in WT and AD samples according to CT-seq and TAB-seq.

**D-E)** Venn diagrams of overlapping 5mC loci obtained by CT-seq between two biological replicates of WT (**D**) and AD (**E**) samples.

**F-G)** Venn diagrams of overlapping 5hmC loci obtained by TAB-seq between two biological replicates of WT (**F**) and AD (**G**) samples.

**H-I)** Frequency distribution of methylation levels determined by CT-seq (**H**) and hydroxymethylation levels determined by TAB-seq (**I**) for each WT and AD sample. The color gradient represents different levels of methylation or hydroxymethylation, ranging from 0-10% (dark blue) to 100% (dark red).


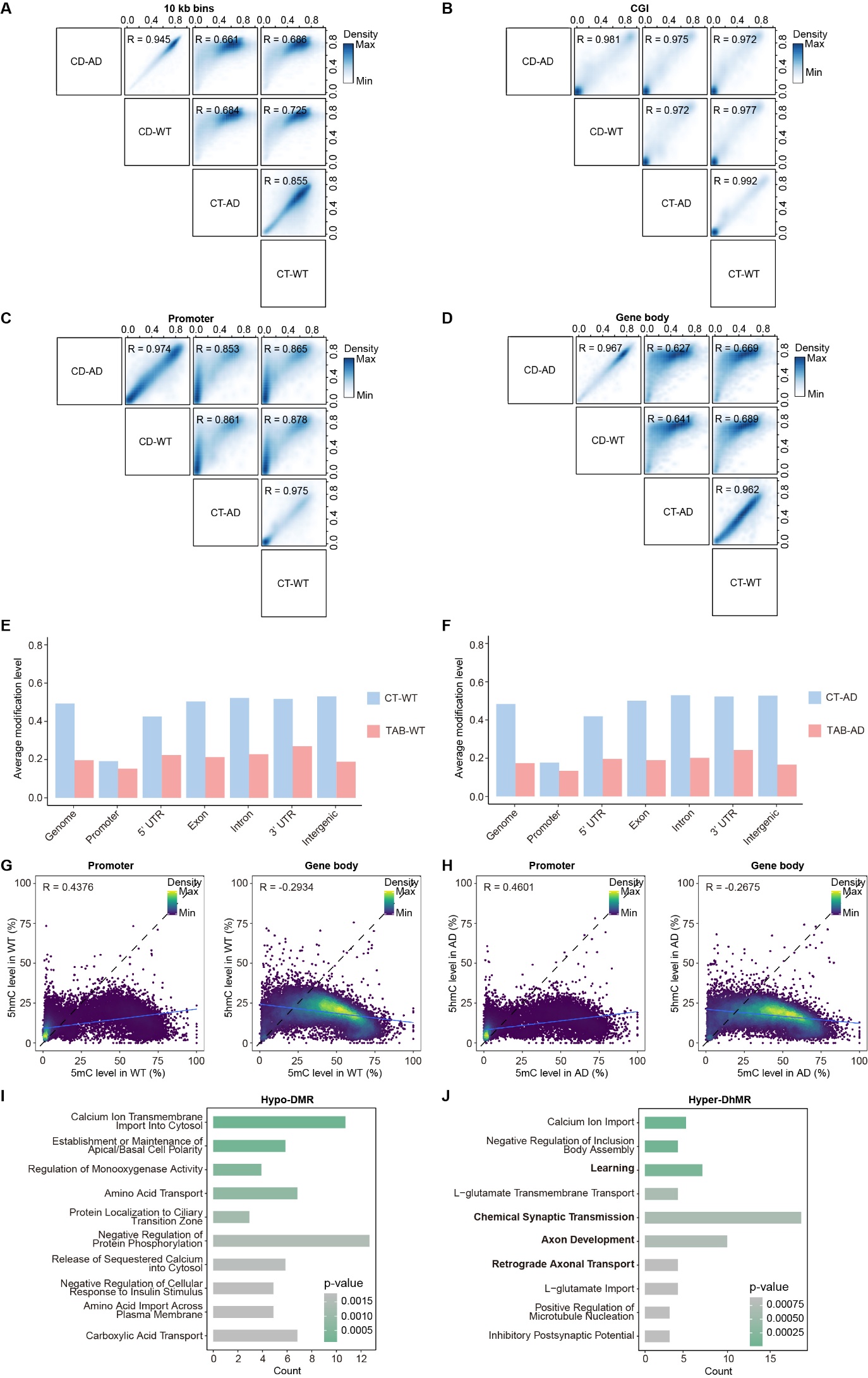


**Figure S10. Consistency of 5mC sequencing data generated from CD-seq and CT-seq.**

**A-D)** Heatmaps showing the Pearson correlation coefficients (R) for 5mC levels determined by CD-seq and CT-seq across various samples in 10 kb bins (**A**), CGIs (**B**), promoters (**C**), and gene bodies (**D**). The samples and sequencing methods are as indicated. Each point represents a 10 kb bin (A), a CGI (B) or a gene (C/D), while the color indicating the point density.

**E-F)** Average 5mC (blue) and 5hmC (red) levels in various genomic regions of WT (**E**) and AD (**F**) samples determined by CT-seq and TAB-seq.

**G-H)** Correlation between 5mC and 5hmC levels in promoters (left) and gene bodies (right) of WT (**G**) and AD (**H**) mice, as determined by CT-seq and TAB-seq. Each point represents a gene, while the color indicating the point density.

**I-J)** Gene ontology analysis of hypo-DMRs from CT-seq (**I**) and hyper-DhMRs from TAB-seq (**J**). The color of each bar represents the *p* value, with darker shades indicating lower *p* values.


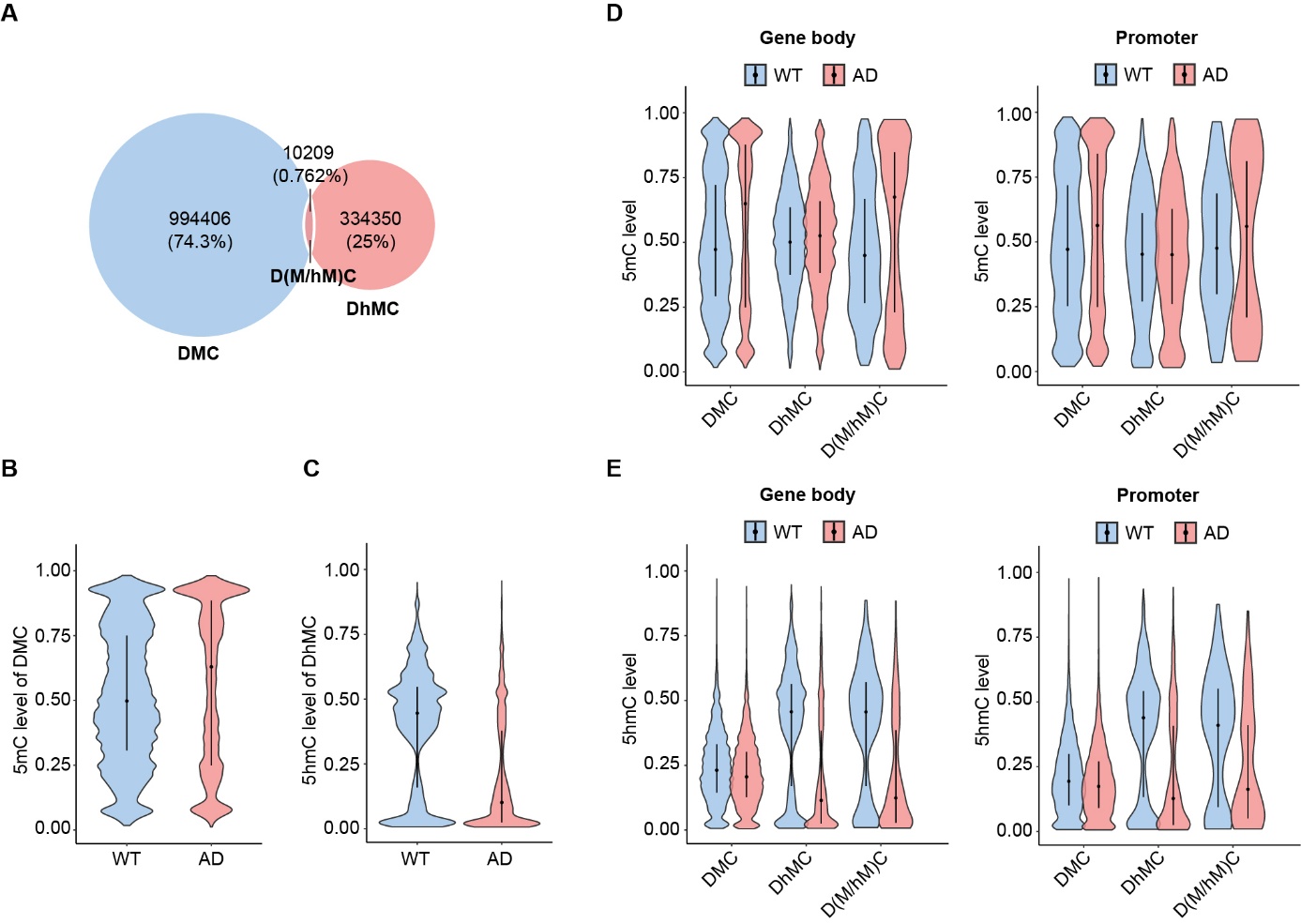


**Figure S11. Independent analysis of 5mC and 5hmC reveals exclusive distribution of DMC and DhMC, as determined by CT-seq and TAB-seq.**

**A)** Overlap analysis between DMC and DhMC determined by CT-seq and TAB-seq. Cytosines in the overlapping region are classified as D(M/hM)C. DMC and DhMC with a *p* value < 0.05 and a mean methylation difference ≥ 0.1 were considered significantly differentially methylated or hydroxymethylated.

**B-C)** Average levels of 5mC (**B**) or 5hmC (**C**) in DMC or DhMC in WT and AD samples determined by CT-seq and TAB-seq. The width of the violin plot represents the frequency of data at each value. The short horizontal line denotes the interquartile range of the data, while the black dot indicates the median.

**D-E)** 5mC (**D**) or 5hmC (**E**) levels within gene bodies (left) and promoters (right) for all DMC, DhMC, and D(M/hM)C in WT (blue) and AD (red). The width of the violin plot represents the frequency of data at each value. The short horizontal line denotes the interquartile range of the data, while the black dot indicates the median.


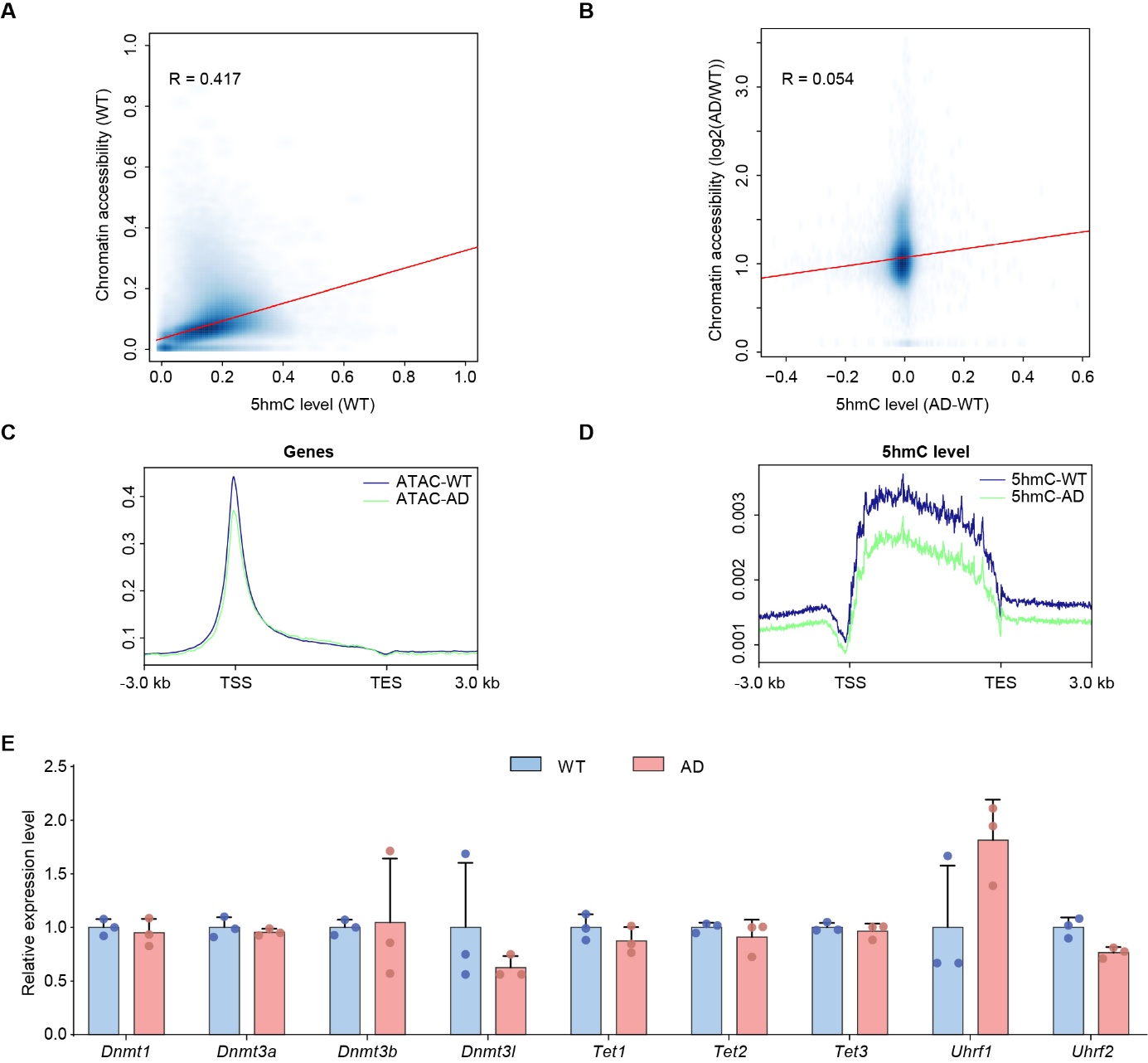


**Figure S12:** 5hmC is positively associated with chromatin accessibility.

**A**) Global correlation between chromatin accessibility and 5hmC levels across the hippocampal genome.

**B**) Correlation between differential chromatin accessibility and 5hmC changes in AD versus WT mice.

**C**) Genome-wide distribution of chromatin accessibility across distinct genomic features.

**D**) Distribution of 5hmC across genomic contexts.

**E)** Relative expression level of DNA methylation-related genes in WT (blue) and AD (red) mice. Expression levels in WT are normalized to 1 (n = 3).
